# The Untranslated Regions of Classic Swine Fever Virus RNA Trigger Apoptosis

**DOI:** 10.1371/journal.pone.0088863

**Published:** 2014-02-12

**Authors:** Wei-Li Hsu, Chung-Lun Chen, Shi-Wei Huang, Chia-Chen Wu, I-Hsuan Chen, Muthukumar Nadar, Yin-Peng Su, Ching-Hsiu Tsai

**Affiliations:** 1 Graduate Institute of Microbiology and Public Health, National Chung Hsing University, Taichung, Taichung, Taiwan; 2 Graduate Institute of Biotechnology, National Chung Hsing University, Taichung, Taiwan; 3 Department of Biotechnology, School of Biotechnology and Health Sciences, Karunya University, Coimbatore, Tamil Nadu, India; University of British Columbia, Canada

## Abstract

*Classical swine fever virus* (CSFV) causes a broad range of disease in pigs, from acute symptoms including high fever and hemorrhages, to chronic disease or unapparent infection, depending on the virus strain. CSFV belongs to the genus Pestivirus of the family *Flaviviridae*. It carries a single-stranded positive-sense RNA genome. An internal ribosomal entry site (IRES) in the 5′ untranslated region (UTR) drives the translation of a single open reading frame encoding a 3898 amino acid long polypeptide chain. The open reading frame is followed by a 3′ UTR comprising four highly structured stem-loops. In the present study, a synthetic RNA composed of the 5′ and 3′ UTRs of the CSFV genome devoid of any viral coding sequence and separated by a luciferase gene cassette (designated 5′UTR-Luc-3′UTR) triggered apoptotic cell death as early as 4 h post-transfection. The apoptosis was measured by DNA laddering analysis, TUNEL assay, annexin-V binding determined by flow cytometry, and by analysis of caspase activation. Contrasting with this, only trace DNA laddering was observed in cells transfected with the individual 5′ or 3′ UTR RNA; even when the 5′ UTR and 3′ UTR were co-transfected as separate RNA molecules, DNA laddering did not reach the level induced by the chimeric 5′UTR-Luc-3′UTR RNA. Interestingly, RNA composed of the 5′UTR and of stem-loop I of the 3′UTR triggered much stronger apoptosis than the 5′ or 3′UTR alone. These results indicate that the 5′ and 3′ UTRs act together *in cis* induce apoptosis. We furthered obtained evidence that the UTR-mediated apoptosis required double-stranded RNA and involved translation shutoff possibly through activation of PKR.

## Introduction

*Classical swine fever virus* (CSFV), belonging to the genus *Pestivirus* in the *Flaviviridae* family, is the causative agent of classical swine fever (CSF), a highly contagious disease that causes serious economic losses to the pig industry. The disease severity is dependeant on the virus strain and the age of pigs. Depletion of lymphocytes is the most significant immunopathological consequence of acute CSFV infection that is attributed to an induction of apoptosis in non-infected bystander cells, possibly via the action of tumor necrosis factor (TNF)-α or interferon (IFN)-α[1], [2], [3].

CSFV is an enveloped virus with a genome consisting of single-stranded, positive-sense RNA of approximately 12.3 kb [4]. The RNA genome carries a single large open reading frame flanked by a 5′ and a 3′ untranslated region (UTR). The open reading frame encodes a 3898-amino acid polyprotein which is processed into 12 mature proteins N^pro^, C, E^rns^, E1, E2, p7, NS2, NS3, NS4A, NS4B, NS5A and NS5B by viral and host proteases [5].

The 5′ and 3′ UTR of CSFV RNA are 374 and 230 nucleotides in length, respectively. The sequences of the 5′ UTR show a high degree of conservation among the *Pestiviruses* CSFV, *Bovine viral diarrhea virus* (BVDV) and *Border disease virus* (BDV). The 5′ UTR forms three structural domains and a pseudoknot with the sequence downstream of the initiation codon, and functions as an internal ribosome entry site (IRES) to allow the initiation of cap-independent translation [6], [7], [8]. It has been shown that the IRES of CSFV recruits the 40 S ribosomal subunit but none of the known initiation factors [7].

In eukaryotes, the poly(A)-binding protein (PABP) and eukaryotic initiation factor 4G (eIF4G) bind to the 3′-poly(A) tail and the 5′-terminal cap, respectively, to form a circular structure. This strategy enhances translation efficiency by recycling ribosome for successive rounds of translation [9], [10]. Similar phenomenon was observed in some RNA viruses such as Hepatitis C virus (HCV) [11]. Circularization of cellular mRNA is mainly mediated by the recruitment of protein factors, by RNA-protein and protein-protein interactions to bridge the 5′ and the 3′ UTRs [12]. HCV uses complementary sequences to form loops or motifs at both ends of its RNA genome, which enables RNA circularization and facilitates translation independent from proteins [13]. Very recently, we demonstrated that the 3′ UTR of CSFV regulates the IRES-dependent translation [14]. In the absence of the 3′ UTR, the translation of a reporter gene was dramatically decreased. The addition of the stem-loops (SL) 2 and 3 (SLII and SLIII) of the 3′ UTR to 5′UTRs upregulated the translation of reporter gene, whereas moving SLI to the end of SLII and SLIII downregulated translation. While studying the effect of the CSFV UTRs on regulation of translation, unexpected cell death was observed in cells transfected with RNA composed of CSFV 5′ and 3′ UTRs. Therefore, the present study was aimed at identifying the requirements of the CSFV UTR to trigger apoptosis, and at exploring the possible mechanisms involved.

## Materials and Methods

### Cells and Viruses

Cells were obtained from Bioresource Collection and Research Center/Food Industry Research and Development Institute of Taiwan. Rabbit kidney 13 (RK-13) cell line (BCRC 60010), HeLa cells (BCRC-60005), and Madin-Darby canine kidney (MDCK) cell line (BCRC 60004) were grown in Dulbecco's modified eagle medium (DMEM); Porcine kidney 15 (PK-15) cell (BCRC 60057), PK-15 cells constitutively expressing CSFV N^pro^ protein fused to green fluorescent protein (GFP) [15], and human embryonic kidney 293 T cell line were grown in Dulbecco's modified eagle medium (DMEM) (GIBCO, Grand Island, NY, USA), supplemented with 1.0 mM sodium pyruvate, 1.5 g/L sodium bicarbonate, 1 unit/ml penicillin G sodium, 100 µg/ml streptomycin sulfate, and 10% fetal calf serum (GIBCO).

### Generation of plasmids or DNA fragments for RNA preparation

The plasmid pALD/L/A contains sequences of 5′ and 3′ UTRs of CSFV [14]. This plasmid was used as template for generating 5′UTR-Luc-3′UTR (the luciferase gene flanked with the 5′ and 3′ UTRs of CSFV) and 5′-Luc RNA (the luciferase gene with 5′ UTR of CSFV). The plasmid (pUC18-ALD-3′UTR) containing the 3′UTR was generated by insertion of the blunt-ended 3′UTR fragment amplified by PCR using the forward primer CSFV ALD 5′(+)T7 +12,058 ( 5′-TAATACGACTCACTATAGGGTATGAGCGCGGGTAA*CCCGGG*ATCTGGA; the *Sma*I site indicated in italics and the T7 promoter sequences underlined), and reverse primer ALD 5′(-) -12,328 (5′ GGGCCGTTAGGAAATTACCTTAGTC) into pUC18 plasmid.

DNA templates for *in vitro* transcription were produced by digestion of the appropriate plasmid with specific restriction enzymes or amplified by PCR. For instance, digestion of pALD/L/A with *Sse8387*I for 5′UTR-Luc-3′UTR RNA and *Age*I for 5′UTR RNA, digestion of pUC18-ALD-3′UTR with *Bam*H I for 3′UTR, digestion of SP6LUC plasmid (Promega, Madison, WI, USA) with *Xmn*I for control luciferase RNA were performed. For *in vitro* transcription of RNA containing the 5′UTR-luciferase fused to one of the four stem loops (i.e. SLI, SLII, SLIII, and SLIV) of 3′UTR, templates were generated by PCR from plasmid pALD/L/SLI, pALD/L/SLII, pALD/L/SLIII, and pALD/L/A [14], using a universal forward primer (5′-GACGTCTAAGAAACCATTATTATC) and the reverse primers: SLI (5′-GGGCCGTTAGGAAATTACCTTA), SLII (5′-CTGTTAAAAATGAGTGTAGTGTGGT), SLIII (5′-TAAATAAATAAATAAATAGTAATAT), SLIV(5′-TAGGGTCCTACTGGCGGGTCCAGAT), respectively.

### *In vitro* transcription

Five micrograms of linearlized DNA was transcribed *in vitro* in a 50 µl reaction containing 150 U of T7 RNA polymerase (or SP6 polymerase for generation of luciferase RNA) (Promega), 40 mM Tris-HCl pH 8.0, 8 mM MgCl_2_, 2 mM spermidine-(HCl)_3_, 10 mM dithiothreitol, 1 mM NTPs, at 37°C for 2 h. DNA template was removed by RNase-free DNase I treatment, and followed by a phenol/chloroform extraction and ethanol precipitation. RNAs were resolved in 1.2% agarose gel and RNA quality was measured by densitometry.

### DNA laddering assay

Approximately 3−4×10^5^cells/well were seeded in 12-well plates with appropriate medium. The culture was replenished the next day before transfection with fresh medium lacking FBS and penicillin/streptomycin. Cells were transfected 0.625 µg RNA/well (unless otherwise stated) using Lipofectamine 2000 transfection reagent (Invitrogen, Carlsbad, California, U.S.A.) according to the manufacture's instructions. Harvested cells were lysed with buffer containing 0.25% EDTA and 0.2% Triton ×100. The cell lysate was then treated with RNase A (70 µg/ml) at 37°C for 1 h followed by protein kinase K (150 µg/ml) at 50°C for 1 h. Subsequently, the cell lysate was extracted with chloroform (once) and phenol/chloroform (twice). After centrifugation, the aqueous phase containing chromosomal DNA was transferred to a new tube and precipitated with ethanol, air dried and dissolved in 30 µl of deionized water. The DNA samples were quantified by NanoVue Plus spectrophotometer (GE Healthcare Life Sciences). DNA laddering was analysed by electrophoresis in 1.2% agarose gel.

### Detection of apoptotic cells by Hoechst staining and TUNEL assay

PK-15 cells seeded in 12-well plate were transfected with luciferase or CSFV RNAs. 18 h post-transfection, cells were fixed with 4% paraformaldehyde for 10 min, washed with PBS, and incubated with 2 µg/ml of Hoechst 33342 (Sigma-Aldrich, St. Louis, MO, USA) for 1 min. The morphology of cells was observed with fluorescence microscopy.

For TUNEL staining, PK-15 cells at density of 1.5×10^5^ cells/well were seeded onto 1.3 mm coverslips in 24-well plates and were transfected with luciferase or CSFV UTR RNAs. At 4, 8, and 18 h post-transfection, cells were washed twice with phosphate-buffered saline (PBS) and then fixed with 4% parafolmaldehyde. TUNEL staining was performed with an *in situ* cell death detection kit (Roche Applied Science, Mannheim, Germany). The staining was observed with fluorescence microscopy.

### Flow cytometric analysis of apoptosis by FITC-annexin V binding assay

PK-15 cells seeded in 12-well plate at density of 4×10^5^/well were transfected with either luciferase or CSFV UTR RNA (0.625 µg/well) and detection of annexin V was conducted using BD Pharmingen FITC Annexin V Apoptosis Detection Kit I. Briefly, cells were harvested by trypsinization 18 h post-transfection, washed with PBS and resuspended in Binding buffer at a density of 1×10^6^/ml. Cells (1×10^5^) were then incubated with 5 µl of in buffer containing FITC annexin V and propidium iodide (PI) followed by flow cytometric analysis.

### Measurement of DNA fragmentation by ELISA

PK-15 cells seeded in 12-well plate were pre-treated with 100 µM of pan-caspase inhibitor Z-VAD-FAM (Calbiochem, Cat. 627610) or DMSO (as solvent control) for 3 h followed by RNA transfection as described previously. DNA fragmentation levels were measured by ELISA using Cell Death Detection ELISAPLUS kit (Roche Applied Science) 18 h post-transfection.

### Luciferase activity assay

Approximately 4×10^5^ of RK cells were first seeded into 12-well plates and then were grown to 90% confluence. Plasmid p2Luc (0.5 µg) alone, or in combination with 0.05 pmole of CSFV 5′UTR-Luc-3′UTR, 5′ UTR, or 3′ UTR RNA diluted with 100 µl of Opti-MEM were cotransfected into RK cells using Lipofectamine (Invitrogen) according to the manufacture's instruction. Cells were harvested 7 h post-transfection for reporter assay. Briefly, transfected cells were washed with PBS, and then lysed with 150 µl of passive lysis buffer followed by a freeze-thaw process. After a centrifugation at 13,000X*g* for 5 min, 20 µl of lysate was mixed with 100 µl of Luciferase Assay reagent (Promega, Cat. E2810), and the firefly luciferase activity was measured by a luminometer (FLUOstar OPTIMA, BMG Labtech, Ortenberg, Germany).

### Western blot analysis

Lysate of RNA transfected cells were separated by 15% SDS-PAGE and electrotransferred to nitrocellulose membrane using Mini Protein III equipment (BioRad, Life science Research Products, CA, USA). The filter was blocked with PBS-T buffer (0.02 M phosphate, 0.15 M NaCl, 0.05% Tween-20) containing 5% skim milk powder at room temperature for 1 h and probed with 1∶1,000 diluted first antibody (Cell Signaling Technology, Inc., Danvers, MA, USA), i.e. rabbit anti-cleaved Caspase-3 (Asp175, #9661S) antibody, mouse anti-eIF-2α (#L57A5), rabbit anti-phosphor-eIF-2α (#ab32157, abcam, Cambridge, UK), or mouse anti-β-actin (from GeneTex Inc, Irvine, CA, USA) at 4°C for overnight. After several washes with PBS, the membrane was then incubated with the secondary antibody, goat anti-rabbit (or mouse) IgG conjugated with horseradish peroxidase (HRP), diluted 1∶10,000 for 1 h at room temperature. Unbound antibodies were removed by PBS washes and the signal was detected using an AEC (Red) Substrate Kit (Invitrogen).

## Results

### Transfection of the CSFV UTR in absence of any viral coding sequences trigger apoptosis

**Figure 1:**
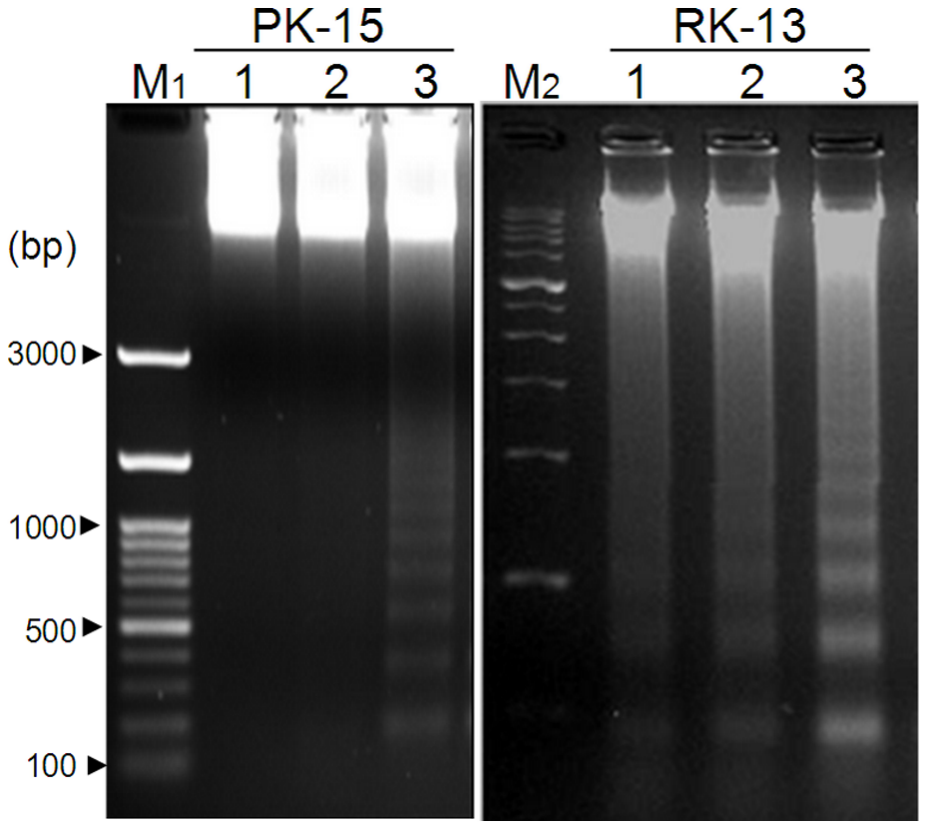
Transfection of CSFV UTR RNA triggers DNA laddering. Porcine kidney (PK-15) or rabbit kidney (RK-13) cells were mock transfected (lane 1), or transfected with *in vitro* transcribed luciferase RNA (lane 2), or chimeric luciferase-CSFV UTR RNA (5′UTR-Luc-3′UTR, lane 3) for 16 h. Total cellular DNA was harvested for the DNA laddering assay. M1, M2, and M were standard DNA markers with various size ranges.

In a previous study addressing the translational regulation of CSFV UTRs, *in vitro* transcribed RNA of which the coding region of CSFV was replaced by a firefly luciferase reporter gene (designated ALD/L/A or 5′UTR-Luc-3′UTR in the present study) was transfected into porcine kidney (PK-15) cells [14]. Unexpectedly, cell death was observed when cells were transfected with the 5′UTR-Luc-3′UTR RNA, which was further explored here. Apoptosis was first investigated by DNA laddering assay in the porcine kidney cells PK-15 and in the rabbit kidney cells RK-13, the two cell lines commonly used to study CSFV. As shown in Fig. 1, a DNA laddering pattern was observed in PK-15 cells transfected with 5′UTR-Luc-3′UTR RNA (lane 3), but not in the control transfections in absence of RNA (lane 1) or with luciferase RNA (lane 2). Similar results were obtained with RK-13 cells, as evidenced by a strong increase of the DNA laddering in the presence of 5′UTR-Luc-3′UTR RNA.

### Characterization of CSFV UTR-induced cell death by terminal transferase deoxyribonucleotidyl dUTP nuclear end labeling (TUNEL) and annexin V binding assays

**Figure 2:**
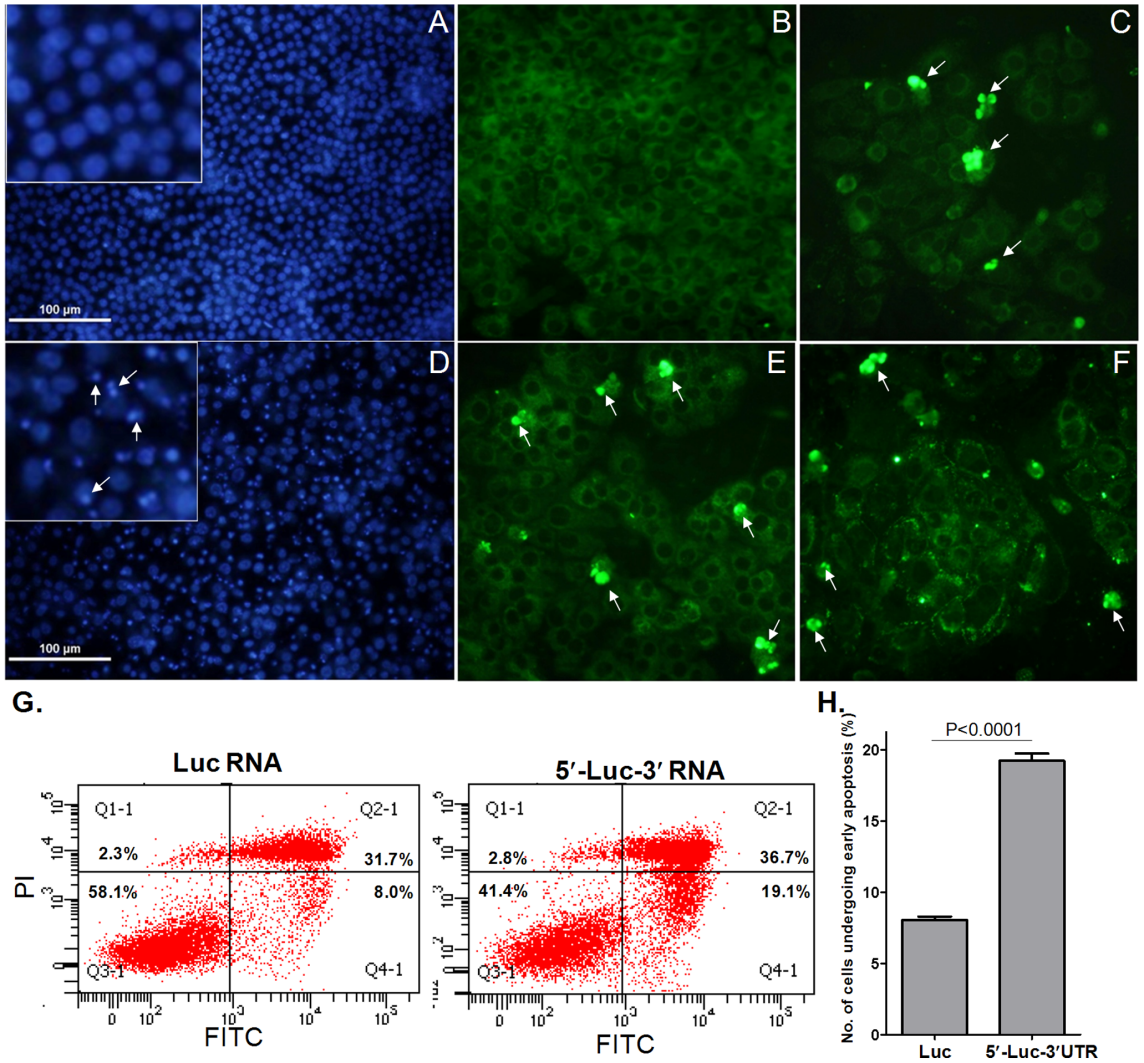
Confirmation of CSFV-UTR-triggered apoptosis using different methods. PK-15 cells were transfected with luciferase RNA (A, B) or chimeric luciferase-CSFV UTR RNA (5′UTR-Luc-3′UTR, C- F). At 4 h (C), 8 h (E), or 18 h (D, F) post-transfection, cells were processed for Hoechest 33342 staining (A, D) or TUNEL assay (B, C, E, F). The inset in panel A and D shows the cells at higher magnification. Arrowheads indicate the apoptotic bodies representing nuclear condensation (D) and the apoptotic cells (C-F). The proportion of cells undergoing apoptosis was also evaluated using an annexin V-binding assay (G). PK-15 cells were transfected with either luciferase RNA or 5′UTR-Luc-3′UTR RNA. Annexin-V-FITC/PI flow cytometry analysis was performed at 18 h post-transfection. Q4-1 indicates the number of cells undergoing early apoptosis. The experiments were done in triplicates and the mean value of Q4-1 was plotted (H). Compared with cells transfected with control RNA, apoptosis increased 2.3 (±0.098) fold (P value <0.0001) in cells transfected with CSFV UTR RNA.

Apoptotic cell death induced by transfection of CSFV UTR RNA was confirmed using alternative analytical approaches to DNA laddering. With Hoechest 33342 staining, nuclear condensation was clearly enhanced in 5′UTR-Luc-3′UTR RNA-transfected cells compared to luciferase RNA-transfected cells (Fig. 2A and D). A clear increase in the number of floating dead cells was observed starting 8 h after transfection (data not shown). Using the TUNEL assay, apoptosis was detected in numerous cells transfected with 5′UTR-Luc-3′UTR RNA as early as 4 h post-transfection (Fig. 2C). There was however no further increase in the number of apoptotic cells at 8 h (Fig. 2E) and 18 h (Fig. 2F) after transfection. This might be due to the loss of cells undergoing apoptosis at late times after transfection. Apoptosis was also monitored by flowcytometry by detecting the exposure of phosphatidylserine residues on the outer plasma membrane through binding of fluorescein isothoicynate (FITC)-conjugated annexin-V. In parallel, dead cells were monitored by propidium iodide (PI) incorporation (Fig. 2G). Consistent with the other assays, a statistically significant 2.3 (±0.098) fold increase in apoptosis was observed 16 h post-transfection in cells transfected with the CSFV UTR RNA 5′UTR-Luc-3′UTR compared with control Luciferase RNA (Fig. 2H).

### CSFV UTRs triggers apoptosis in a caspase-dependent manner

**Figure 3:**
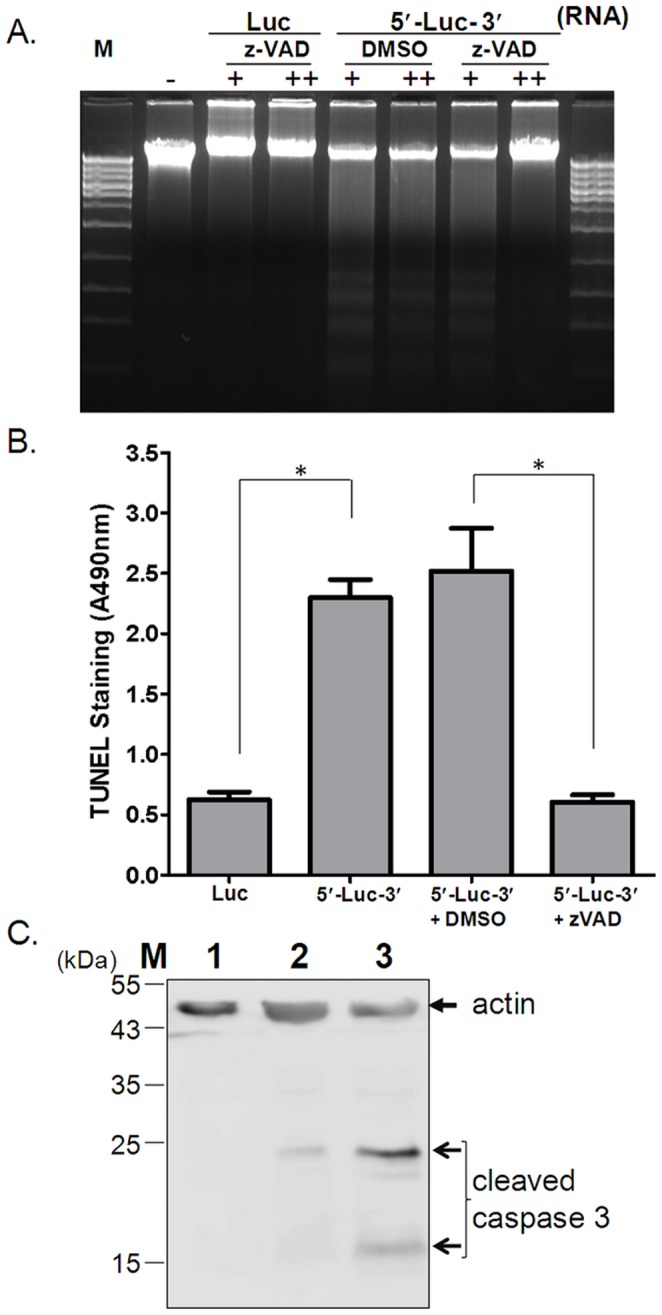
Apoptosis triggered by CSFV UTR RNA is caspase dependent. PK-15 cells were treated with two different concentrations (+: 20 μM; ++:100 μM) of the pan-caspase inhibitor z-VAD-FMK (z-VAD) or with equal volume of DMSO (the solvent control), or were left untreated (−) for 7 h prior to transfection of 5′UTR-Luc-3′UTR RNA (CSFV-UTR) or luciferase RNA. DNA was extracted for DNA laddering analysis (A), and total protein from transfected cells was processed for TUNEL ELISA (B). Activation of caspase 3 was analysed by Western blot (C). PK-15 cells were transfected with luciferase RNA (lane 2), CSFV UTR RNA (lane 3), or untransfected (lane 1). M: DNA size marker. Asterisks indicate p value <0.05.

Apoptosis is initiated by the activation of a number of caspases [17]. The pan-caspase inhibitor Z-VAD-FMK was employed to further confirm CSFV UTR-mediated programmed cell death. As shown in Fig. 3A, DNA laddering was observed in CSFV 5′UTR-Luc-3′UTR-transfected cells treated with DMSO (solvent control), whereas it was blocked by the addition of 100 µM of Z-VAD-FMK. However treatment with lower a concentration (20 µM) of Z-VAD-FMK did not effectively inhibit the DNA laddering. Caspase-dependent apoptosis was confirmed with TUNEL ELISA. Addition of Z-VAD-FMK significantly decreased the TUNEL signal (Fig. 3B), consistent with the previous results. As caspase-3 is the executioner of apoptosis, the activation of caspase 3 was determined by Western blot analysis. Cleavage of caspase 3 was observed in cells transfected with CSFV 5′UTR-Luc-3′UTR RNA, but not in the control transfections (Fig. 3C). Collectively, DNA laddering, TUNEL assay, formation of apoptotic bodies (Hoechest staining), cleavage of caspase 3, and annexin V binding demonstrate that the CSFV UTRs stimulate transfected cells to undergo apoptosis.

### Efficient induction of apoptosis by CSFV UTR results from synergistic effects of the 5′ and 3′ UTRs *in cis*

**Figure 4:**
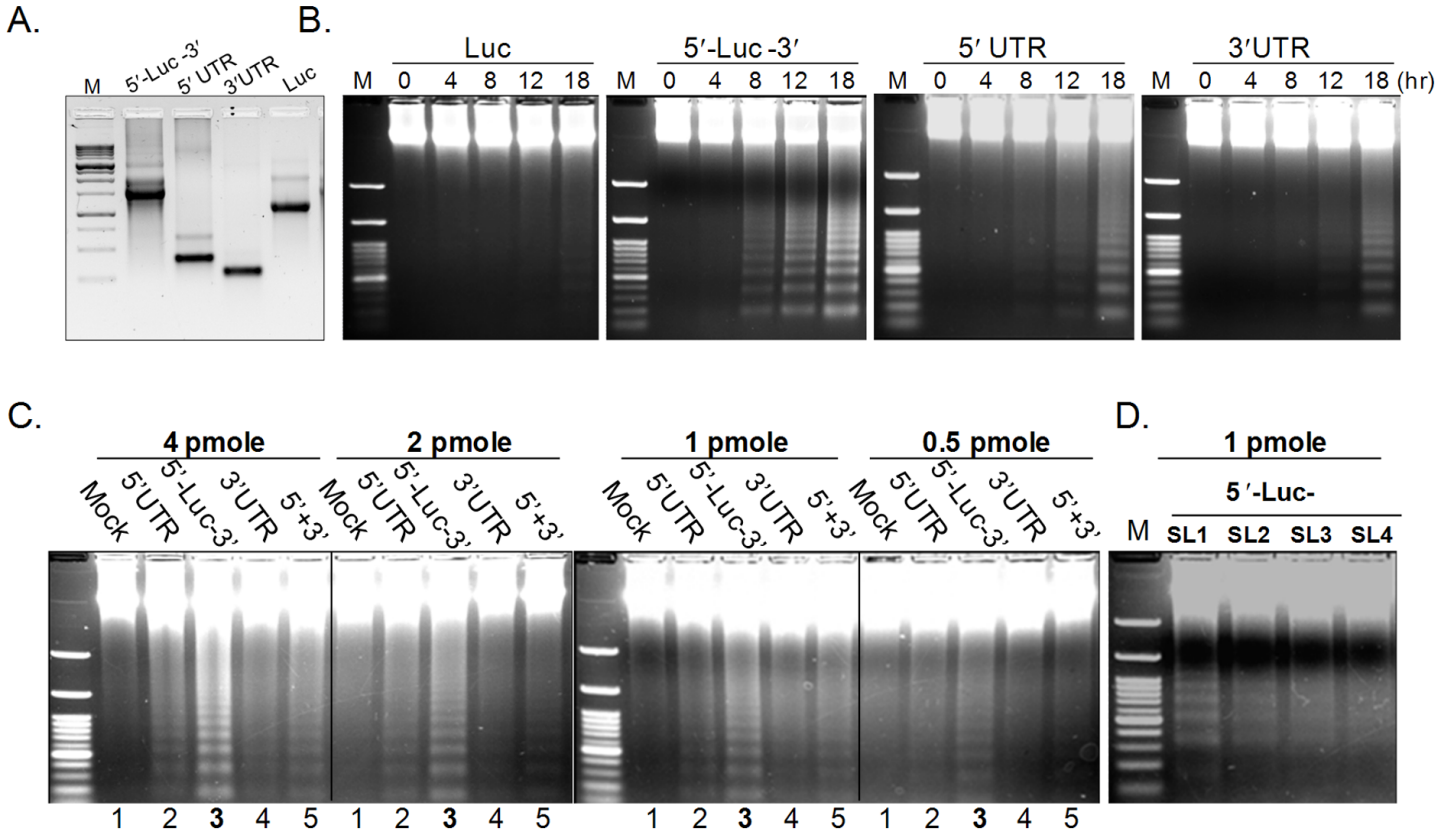
The 5′ and 3′ UTRs are most efficient at inducing apoptosis when present on the same molecule. *In vitro* transcribed 5′UTR-Luc-3′UTR (5′-Luc-3′), 5′UTR, 3′UTR and luciferase RNA (Luc) were analysed by agarose gel electrophoresis (A) and used to transfect PK-15 cells. At 4, 8, 12, 18 h post-transfection, total DNA was harvested for the DNA laddering assay (B). The cells were also transfected with different amounts (0.5, 1, 2, 4 pmole) of 5′UTR-Luc-3′UTR, 5′UTR, 3′UTR, or of a mixture of the individual 5′UTR and 3′UTR (5′+3′) RNA (C). The contribution of each stem loop (SL) of the 3′UTR (as indicated on the top of the gel) to aptoptosis was assessed by transfection of PK-15 cells with 1 pmole of 5′UTR alone (−) or of chimeric RNA consisting of 5′UTR-Luc fused to either of SL1, SL2, SL3, or SL4 of the 3′ UTR (D). In (C) and (D), total DNA was harvested 18 h post-transfection for the DNA laddering assay.

Having confirmed that the CSFV UTRs could induce apoptosis, it was determined which UTR structures possessed the highest capacity to induce apoptosis. To this end, RNA composed of the two UTRs on the same molecule (5′UTR-Luc-3′UTR), of the 5′UTR or the 3′UTR alone, or of control Luc RNA were transcribed *in vitro* (Fig 4A) and used to transfect cells for different times and at different concentrations (Fig. 4B-C). Apoptosis was analysed by DNA laddering assay. The strongest DNA fragmentation was observed in the cells transfected with the 5′UTR-Luc-3′UTR as early as 8 h post-transfection, with an increase over time (Fig. 4B). DNA laddering was barely visible with the 5′UTR and 3′UTR RNA alone at 8 and 12 h post-transfection. This shows that the 5′UTR-Luc-3′UTR RNA is more potent at inducing apoptosis than the 5′UTR or 3′UTR alone at the same molarities, suggesting synergistic effects of the CSFV UTRs on apoptosis. In order elaborate on this, PK-15 cells were transfected with different molarities of 5′UTR-Luc-3′UTR, of 5′UTR and 3′UTR alone, or of a mixture of the 5′UTR and the 3′UTR (5′+3′). As shown in Fig. 4C, among the four different RNAs transfected, 5′UTR-Luc-3′UTR RNA induced consistently the strongest DNA fragmentation in a dose-dependent manner. Transfection of the individual 5′UTR and 3′UTR RNA separately (Fig. 4C lanes 2 and 4) or as a mixture (Fig. 4C lane 5) triggered much less DNA fragmentation than the 5′UTR-Luc-3′UTR RNA (lane 3). These data suggest that the 5′UTR and the 3′UTR of CSFV form a higher order structure *in cis* that might facilitate the induction of apoptosis.

### SLI of the 3′UTR together with the 5′UTR function *in cis* to induce apoptosis

For HCV and CSFV, it was reported that the interaction of the respective 5′ UTR containing the IRES structure with the 3′UTR 5BSL3.2 or SLI-IV of HCV or CSFV respectively, regulated viral protein translation [11], [14]. Similarly, the interaction of the 5′ and 3′ UTRs may also form structures or complexes that induce apoptosis. The data above suggests that CSFV UTR-induced apoptosis results from a cross-talk between the 5′UTR and the 3′UTR *in cis*. Therefore it was investigated which part of the 3′ UTR needs to be associated with the 5′UTR on the same molecule to induce apoptosis. To this end, four plasmids were constructed to produce RNA containing 5′-Luc with 3′-terminal fusion of either of the individual SL sequences SLI, -II, -III, or -IV. The different RNA was synthesized *in vitro* and their capacity to trigger apoptosis were analysed in PK-15-cells. Only the transfection of the 5′-Luc-SLI RNA induced clear DNA fragmentation (Fig. 4D). Altogether, these results indicate that an intra-molecular interaction of SLI of the 3′ UTR with 5′ UTR is required for the induction of apoptosis CSFV UTR structures.

### UTR RNA-induced apoptosis is mediated by double-stranded RNA, independently of the triphosphate moiety

**Figure 5:**
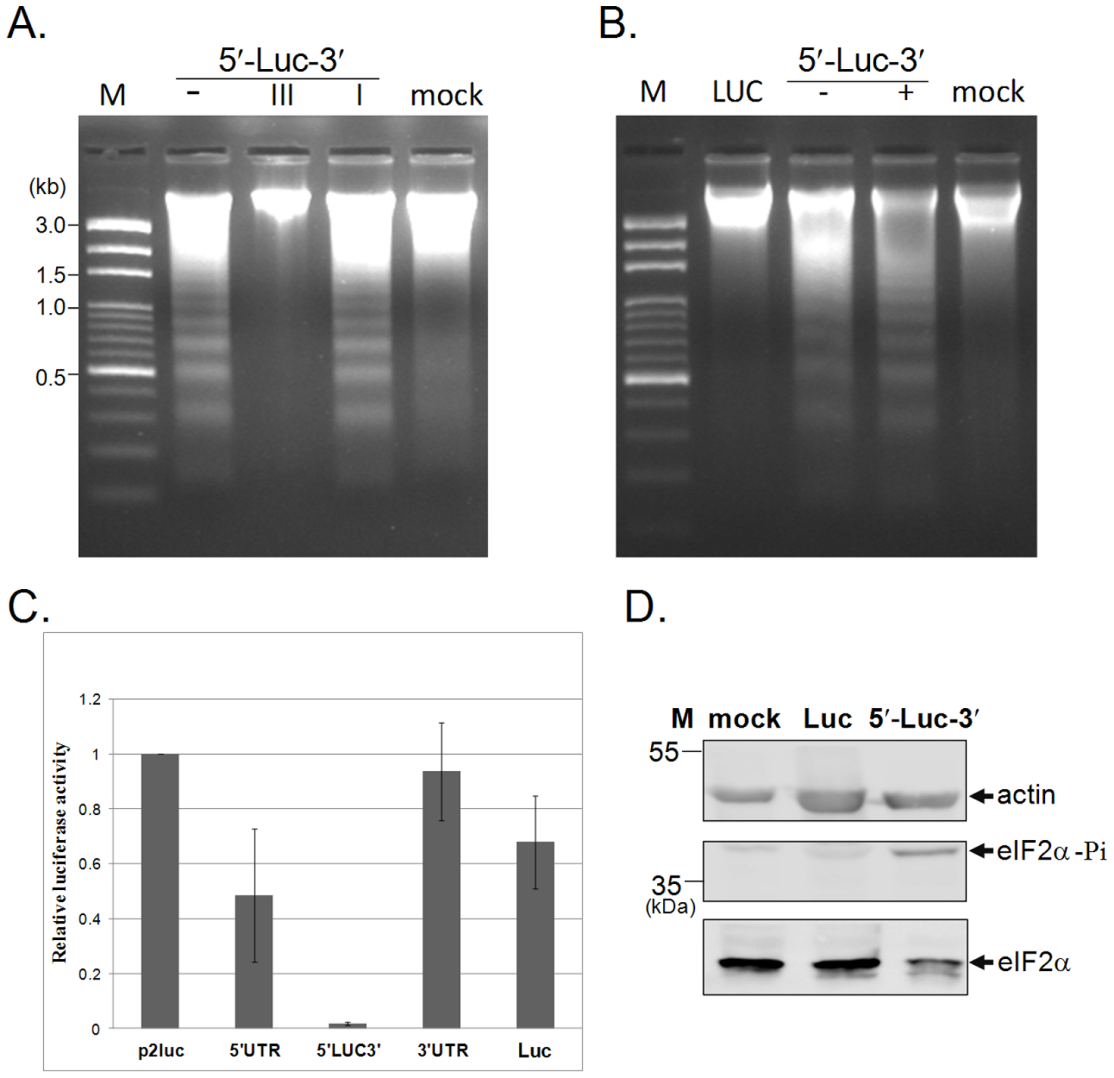
UTR RNA-induced apoptosis is mediated by double-stranded RNA and independent on the triphosphate moiety. (A) Double-stranded structures contribute to the UTR RNA-triggered apoptosis. PK-15 cells were left untreated (mock), or transfected with *in vitro* transcribed untreated (−), RNaseIII-treated (III), or RNaseI-treated (I) 5′UTR-Luc-3′UTR RNA (5′-Luc-3′) for 16 h prior to DNA laddering assay. (B) PK-15 cells were mock-transfected (mock), or transfected with *in vitro* transcribed luciferase RNA (Luc), or with calf intestinal phosphotase-treated (+) or untreated (−) 5′UTR-Luc-3′UTR RNA (5′-Luc-3′) for 16 h prior to DNA laddering assay. M: DNA size marker. (C) The degree of apoptosis correlates with the level of suppression of translation. PK-15 cells were transfected with the p2luc reporter plasmid (0.5 µg) alone, or in combination with 0.05 pmole of 5′UTR, 5′UTR-Luc-3′UTR (5′LUC3′), 3′UTR, or control firefly luciferase (Luc) RNA. The expression of *Renilla* luciferase (from p2luc plasmid) was quantified 7 h post-transfection. (D) Activation of PKR is responsible for the shutoff of protein synthesis. Total protein of mock-transfected PK-15 cells (lane 1) or of cells transfected with Luc RNA (lane 2) or 5′UTR-Luc-3′UTR RNA (lane 3) was resolved by SDS-PAGE. The presence of phosporylated eIF-2α (eIF-2α–Pi) was determined by Western blot analysis.

The double-stranded RNA (dsRNA) generated during BVDV infection induces apoptosis that contributes to viral cytopathogenicity [18]. In order to examine whether the double-stranded RNA structures of the CSFV UTR elements were sufficient to induce apoptosis in transfected cells, *in vitro* transcribed 5′UTR-Luc-3′UTR RNA was treated with RNase I or RNase III to degrade single or double-stranded RNA, respectively. Cells transfected with mock-treated or RNase I-treated RNA induced DNA fragmentation, while RNase III treatment prevented apoptosis induction (Fig. 5A), indicating that the apoptosis is induced by dsRNA structures (the stem-loops in the UTRs or the long-distance interaction by base-paring between 5′ and 3′ UTRs). Addition of the RNA to the cells without transfection reagents did not cause apoptosis (data not shown). The pattern recognition receptor retinoic acid-inducible gene product I (RIG-I) selectively detects cytoplasmic 5′-triphosphorylated single- and double-stranded viral RNA, inducing proteins of the interferon pathway and triggering apoptosis as part of the innate immune response [19]. Therefore the role of the 5′ triphosphates in the induction of apoptosis by CSFV UTR was analysed. Removal of the 5′-phosphate from 5′UTR-Luc-3′UTR RNA by calf intestine phosphotase (CIP) treatment did not affect induction of apoptosis in transfected cells (Fig. 5B). Thus, the CSFV 5′ and 3′UTR together can trigger apoptosis through dsRNA structures independently of 5′ triphosphates.

### Transfection of viral UTRs represses host cell translation

Since dsRNA can trigger apoptosis through activation of PKR, resulting in the inhibition of translation initiation [20], it was investigated whether CSFV UTR-induced apoptosis caused translation shutoff. Translation of cellular proteins was monitored using a luciferase reporter gene assay. Considering the fact that the onset of apoptosis might interfere with the interpretation of the dsRNA-mediated effects on protein expression, minimal quantities of UTR RNA (0.05 pmole) were used in this assay, and the reporter gene expression was detected before the onset of cell death (i.e. at 7 h post-transfection). In the presence of 5′UTR-Luc-3′UTR, luciferase activity was barely detectable (Fig. 5C). Interestingly, the severity of apoptosis paralleled with the level of translation inhibition. In order to further determine whether the shutoff of protein synthesis is due to the activation of PKR signalling, eIF-2α phosphorylation was analysed. As shown in Fig. 5D, the level of eIF-2α phosphorylation was much higher in cells transfected with 5′UTR-Luc-3′UTR RNA than in cells transfected with control Luc RNA, indicating the correlation of CSFV UTR RNA-induced apoptosis with shutoff of translation.

### Apoptosis triggered by CSFV UTR structures is not suppressed by N^pro^

**Figure 6:**
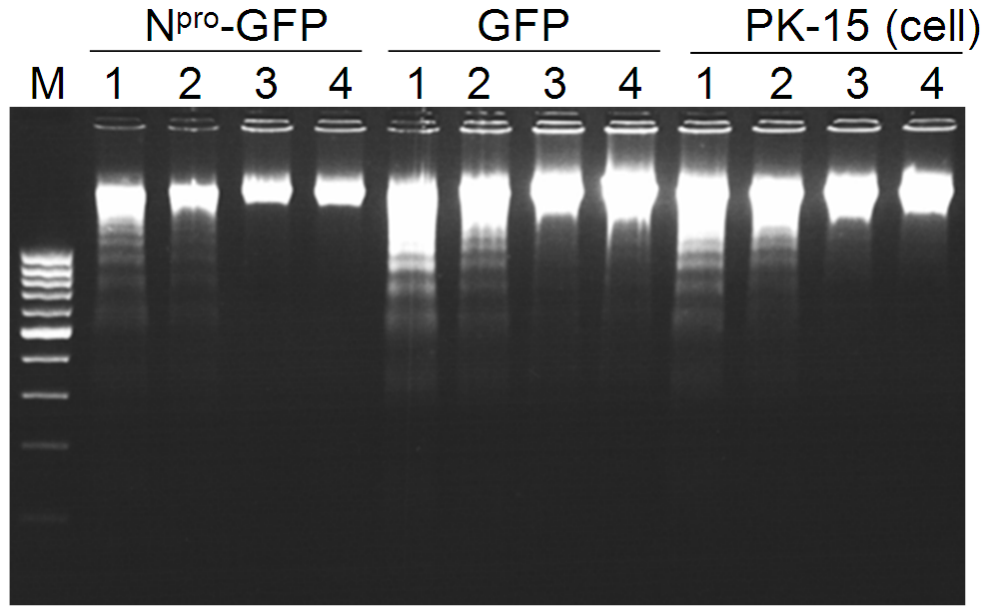
Apoptosis triggered by 5′UTR-Luc-3′UTR RNA is not inhibited by the viral N^pro^ protein. PK-15 cells stably expressing N**^pro^**-GFP or GFP, and the parent PK-15 cells were transfected with *in vitro* transcribed 5′UTR-Luc-3′UTR RNA (lane 1), luciferase RNA (lane 2), or mock transfected (lane 3) or left untreated (lane 4). After 23 h, total DNA was harvested for the DNA laddering assay. M: standard DNA marker.

It was shown that the viral nonstructural protein N^pro^ can suppress apoptosis induced by the synthetic dsRNA pIpC in porcine kidney cell SK-6 [15], [21]. Based on this, we explored whether N^pro^ can inhibit CSFV UTR RNA-mediated apoptosis. To this end, PK-15 cells or PK-15 cell clones stably expressing N^pro^-GFP or GFP alone described previously [15] were transfected with 5′UTR-Luc-3′UTR and control RNA. No obvious difference in DNA laddering induced by 5′UTR-Luc-3′UTR was observed in cells expressing N^pro^ and in control cells (Fig 6). This shows that the N^pro^ protein alone cannot inhibit apoptosis induced by the CSFV UTR structures.

## Discussion

There are several lines of evidences indicating that CSFV infection induces apoptosis in lymphocytes directly and indirectly [1], [2], [22]. Stimulation of pro-inflammatory cytokines (e.g. TNF-α) and viral NS3 expression have been proposed to be involved in the induction of apoptosis and pathogenicity of classic swine fever [1], [23]. In the context of BVDV infection, apoptosis was attributed to dsRNA structures formed during viral RNA replication. The level of dsRNA was positively related to cytopathogenicity of BVDV [18]. In this report we show for the first time that CSFV UTR RNA devoid of any viral proteins can trigger apoptosis. Internucleosomal DNA fragmentation, visible as DNA laddering pattern in gel electrophoresis, is a major marker of apoptosis. However DNA laddering is not detectable in all cell types undergoing apoptosis, and is also not necessarily associated with apoptosis in certain cell types [16], [24], [25]. For these reasons, apoptosis mediated by CSFV UTRs was investigated by several independent methods, including TUNEL assay, formation of apoptotic bodies (Hoechest staining), and annexin V binding. Furthermore, as the programmed cell death is executed by a hierarchy of activated caspases [26], the involvement of caspases was also examined. DNA fragmentation was reverted by treatment with the pan-caspase inhibitor zVAD-FMK, and processing of caspase-3, the effector caspase, was detected in cells transfected with CSFV UTR RNA.

Virus-induced apoptosis and type I IFN synthesis are early host defence mechanisms against viruses that may be triggered and executed through a common pathway [27]; In this context, dsRNA is an important viral trigger that can activate PKR, leading to apoptosis through inhibition of translation in virus-infected cells. In the present study, apoptosis was detected when CSFV UTR RNA was delivered by transfection, indicating that intracellular CSFV UTR RNAs can trigger apoptosis. Furthermore, the severity of apoptosis correlated with the degree of translation inhibition as evidenced by reporter assay and status of eIF-2a phosphorylation (Fig 5C, D). It is very likely that the shutoff of reporter gene expression and induction of apoptosis were both triggered by dsRNA molecules. This was supported by the fact that transfection of 5′UTR-Luc-3′UTR RNA treated with RNase I which degrades single-stranded RNA and leaves double-stranded RNA intact, induced DNA fragmentation to a similar extent than untreated 5′UTR-Luc-3′UTR RNA. Accumulation of dsRNA is found in cells infected with ssRNA or dsRNA viruses as well as DNA viruses [28], [29]. The dsRNA structures result typically from duplexes of complementary strands formed during replication and transcription as well as from secondary structures within ssRNA molecules [30]. In response to virus infections, type I interferons (IFNs) can be activated through the engagement of viral danger signals such as dsRNA and ssRNA sensed by Toll-like receptors (TLRs) and RIG-I-like helicases (RLHs) [31]. RIG-I detects cytoplasmic viral dsRNA or 5′-triphosphorylated ssRNA leading to activation of signalling pathways that mediate type I IFN production. In the present study, DNA fragmentation induced by 5′UTR-Luc-3′UTR RNA was independent of the presence of triphosphates (Fig. 5B), which does however not exclude the engagement of RIG-I.

Although some viruses use apoptosis to destroy infected cells allowing the virus to spread, excessive cell death would prevent the progression of virus infection. Hence, viruses evolved strategies to subvert or delay the onset of cell death following infection. Several reports show that *Pestiviruses* counteract pro-apoptotic effects [21], [32], [33]. For CSFV it was shown that the viral nonstructural protein N^pro^, the first viral protein encoded by the viral genome [34], plays a significant role in suppressing the pIpC-induced apoptosis in the porcine kidney cell line SK-6 [15], [21]. Very recently the inhibition of apoptosis by N^pro^ was proposed to function through the anti-apoptotic protein HAX-1 (HS-1-associated protein X-1) that interacts with the C terminus of N^pro^ [35]. Interestingly, in the present study, apoptosis induced by the CSFV UTR RNA was not suppressed by N^pro^ (Fig 6). This suggests that other viral protein(s) are required for efficient inhibition of apoptosis mediated by CSFV UTR RNA. There is evidence that E^rns^, a viral surface protein, does also contribute to CSFV-mediated inhibition of apoptosis: CSFV repressed caspase 3 activation in pIpC-treated cells, which was reverted in the absence of either N^pro^ or E^rns^ [36]. Furthermore, non-structural protein NS2 was shown to interfere in the apoptotic process under certain circumstances. Stable expression of CSFV NS2 protein upregulated the expression of the anti-apoptotic protein Bcl-2 that contributes to resistance to apoptosis induced by the proteasome inhibitor MG132 in swine umbilical vein endothelial cell line [37]. It was proposed that CSFV infection blocks apoptotic signalling at multiple levels, including at the caspase-8 and the mitochondrial checkpoints through the action of different viral proteins [36]. Whether these proteins would work synergistically in suppressing CSFV UTR RNA induced-apoptosis requires further investigation.

Transfected RNA carrying both, the 5′ and 3′ UTR on the same RNA molecule induced much severe apoptosis than the 5′UTR or the 3′UTR alone at the same molarity (Fig. 4B). These results suggest that an interaction between the 5′UTR and the 3′UTR is required *in cis* for the induction of apoptosis. SLI of the 3′UTR played a major role in this context (Fig. 4C). This is related to our previous results showing that SLI of the CSFV 3′UTR repressed expression of a reporter gene, which was further mapped to the hexamer CGGCCC of the 3′ end of SLI possibly forming a base pair with the sequence in the IRES IIId1 of 5′ UTR, the 40 S ribosomal subunit binding site [14]. These results support a possible interaction of SLI of 3′UTR with 5′UTR RNA being involved in the induction of apoptosis.

In summary, it was shown by several independent experimental approaches that the CSFV UTR RNA can trigger apoptosis in transfected cells. Importantly, to induce apoptosis the 5′ and 3′ UTR RNA have to function together *in cis* but not *in trans*, possibly via interaction of the 5′UTR with SL1 of the 3′ UTR. In this context, the double-stranded structures of the CSFV UTRs are sufficient to trigger apoptosis. Finally, the UTR mediated apoptosis is related to suppression of translation involving the phosphorylation of eIF-2α.

## References

[1] Choi C, Hwang KK, Chae C (2004) Classical swine fever virus induces tumor necrosis factor-alpha and lymphocyte apoptosis. Arch Virol 149: 875–889. 15098104 10.1007/s00705-003-0275-6

[2] Summerfield A, Knotig SM, McCullough KC (1998) Lymphocyte apoptosis during classical swine fever: implication of activation-induced cell death. J Virol 72: 1853–1861. 9499036 10.1128/jvi.72.3.1853-1861.1998 PMC109475

[3] Renson P, Blanchard Y, Le Dimna M, Felix H, Cariolet R, et al (2010) Acute induction of cell death-related IFN stimulated genes (ISG) differentiates highly from moderately virulent CSFV strains. Vet Res 41: 7. 19793538 10.1051/vetres/2009055 PMC2775166

[4] Meyers G, Rumenapf T, Thiel HJ (1989) Molecular cloning and nucleotide sequence of the genome of hog cholera virus. Virology 171: 555–567. 2763466 10.1016/0042-6822(89)90625-9

[5] Meyers G, Tautz N, Becher P, Thiel HJ, Kummerer BM (1996) Recovery of cytopathogenic and noncytopathogenic bovine viral diarrhea viruses from cDNA constructs. J Virol 70: 8606–8613. 8970985 10.1128/jvi.70.12.8606-8613.1996 PMC190953

[6] Chon SK, Perez DR, Donis RO (1998) Genetic analysis of the internal ribosome entry segment of bovine viral diarrhea virus. Virology 251: 370–382. 9837801 10.1006/viro.1998.9425

[7] Kolupaeva VG, Pestova TV, Hellen CU (2000) Ribosomal binding to the internal ribosomal entry site of classical swine fever virus. RNA 6: 1791–1807. 11142379 10.1017/s1355838200000662 PMC1370049

[8] Rijnbrand R, van der Straaten T, van Rijn PA, Spaan WJ, Bredenbeek PJ (1997) Internal entry of ribosomes is directed by the 5′ noncoding region of classical swine fever virus and is dependent on the presence of an RNA pseudoknot upstream of the initiation codon. J Virol 71: 451–457. 8985370 10.1128/jvi.71.1.451-457.1997 PMC191071

[9] Kahvejian A, Svitkin YV, Sukarieh R, M′Boutchou MN, Sonenberg N (2005) Mammalian poly(A)-binding protein is a eukaryotic translation initiation factor, which acts via multiple mechanisms. Genes Dev 19: 104–113. 15630022 10.1101/gad.1262905 PMC540229

[10] Tarun SZ Jr, Sachs AB (1996) Association of the yeast poly(A) tail binding protein with translation initiation factor eIF-4G. EMBO J 15: 7168–7177. 9003792 PMC452544

[11] Romero-Lopez C, Berzal-Herranz A (2009) A long-range RNA-RNA interaction between the 5′ and 3′ ends of the HCV genome. RNA 15: 1740–1752. 19605533 10.1261/rna.1680809 PMC2743058

[12] Sachs AB, Sarnow P, Hentze MW (1997) Starting at the beginning, middle, and end: translation initiation in eukaryotes. Cell 89: 831–838. 9200601 10.1016/s0092-8674(00)80268-8

[13] Harris D, Zhang Z, Chaubey B, Pandey VN (2006) Identification of cellular factors associated with the 3′-nontranslated region of the hepatitis C virus genome. Mol Cell Proteomics 5: 1006–1018. 16500930 10.1074/mcp.M500429-MCP200

[14] Huang SW, Chan MY, Hsu WL, Huang CC, Tsai CH (2012) The 3′-terminal hexamer sequence of Classical swine fever virus RNA plays a role in negatively regulating the IRES-mediated translation. PLoS One 7: e33764. 22432046 10.1371/journal.pone.0033764 PMC3303849

[15] Ruggli N, Bird BH, Liu L, Bauhofer O, Tratschin JD, et al (2005) N(pro) of classical swine fever virus is an antagonist of double-stranded RNA-mediated apoptosis and IFN-alpha/beta induction. Virology 340: 265–276. 16043207 10.1016/j.virol.2005.06.033

[16] Tomei LD, Shapiro JP, Cope FO (1993) Apoptosis in C3H/10T1/2 mouse embryonic cells: evidence for internucleosomal DNA modification in the absence of double-strand cleavage. Proc Natl Acad Sci U S A 90: 853–857. 8430096 10.1073/pnas.90.3.853 PMC45768

[17] Elmore S (2007) Apoptosis: a review of programmed cell death. Toxicol Pathol 35: 495–516. 17562483 10.1080/01926230701320337 PMC2117903

[18] Yamane D, Kato K, Tohya Y, Akashi H (2006) The double-stranded RNA-induced apoptosis pathway is involved in the cytopathogenicity of cytopathogenic Bovine viral diarrhea virus. J Gen Virol 87: 2961–2970. 16963755 10.1099/vir.0.81820-0

[19] Besch R, Poeck H, Hohenauer T, Senft D, Hacker G, et al (2009) Proapoptotic signaling induced by RIG-I and MDA-5 results in type I interferon-independent apoptosis in human melanoma cells. J Clin Invest 119: 2399–2411. 19620789 10.1172/JCI37155 PMC2719920

[20] Garcia MA, Meurs EF, Esteban M (2007) The dsRNA protein kinase PKR: virus and cell control. Biochimie 89: 799–811. 17451862 10.1016/j.biochi.2007.03.001

[21] Ruggli N, Tratschin JD, Schweizer M, McCullough KC, Hofmann MA, et al (2003) Classical swine fever virus interferes with cellular antiviral defense: evidence for a novel function of N(pro). J Virol 77: 7645–7654. 12805464 10.1128/JVI.77.13.7645-7654.2003 PMC164809

[22] Summerfield A, Zingle K, Inumaru S, McCullough KC (2001) Induction of apoptosis in bone marrow neutrophil-lineage cells by classical swine fever virus. J Gen Virol 82: 1309–1318. 11369874 10.1099/0022-1317-82-6-1309

[23] Xu H, Hong HX, Zhang YM, Guo KK, Deng XM, et al (2007) Cytopathic effect of classical swine fever virus NS3 protein on PK-15 cells. Intervirology 50: 433–438. 18204288 10.1159/000113467

[24] Cohen GM, Sun XM, Snowden RT, Dinsdale D, Skilleter DN (1992) Key morphological features of apoptosis may occur in the absence of internucleosomal DNA fragmentation. Biochem J 286 ( Pt 2): 331–334. 10.1042/bj2860331 PMC1132900 1530564

[25] Linfert DR, Chen C, Ma L, Lai T, Tsongalis GJ (1997) Internucleosomal DNA fragmentation in apoptotic myocytes. Clin Chem 43: 2431–2434. 9439471

[26] Cohen GM (1997) Caspases: the executioners of apoptosis. Biochem J 326 ( Pt 1): 1–16. 10.1042/bj3260001 PMC1218630 9337844

[27] Pindel A, Sadler A (2011) The role of protein kinase R in the interferon response. J Interferon Cytokine Res 31: 59–70. 21166592 10.1089/jir.2010.0099

[28] Weber F, Wagner V, Rasmussen SB, Hartmann R, Paludan SR (2006) Double-stranded RNA is produced by positive-strand RNA viruses and DNA viruses but not in detectable amounts by negative-strand RNA viruses. J Virol 80: 5059–5064. 16641297 10.1128/JVI.80.10.5059-5064.2006 PMC1472073

[29] Lee JY, Marshall JA, Bowden DS (1994) Characterization of rubella virus replication complexes using antibodies to double-stranded RNA. Virology 200: 307–312. 8128633 10.1006/viro.1994.1192

[30] Majde JA (2000) Viral double-stranded RNA, cytokines, and the flu. J Interferon Cytokine Res 20: 259–272. 10762073 10.1089/107999000312397

[31] Takeuchi O, Akira S (2009) Innate immunity to virus infection. Immunol Rev 227: 75–86. 19120477 10.1111/j.1600-065X.2008.00737.x PMC5489343

[32] Bensaude E, Turner JL, Wakeley PR, Sweetman DA, Pardieu C, et al (2004) Classical swine fever virus induces proinflammatory cytokines and tissue factor expression and inhibits apoptosis and interferon synthesis during the establishment of long-term infection of porcine vascular endothelial cells. J Gen Virol 85: 1029–1037. 15039545 10.1099/vir.0.19637-0

[33] Schweizer M, Peterhans E (2001) Noncytopathic bovine viral diarrhea virus inhibits double-stranded RNA-induced apoptosis and interferon synthesis. J Virol 75: 4692–4698. 11312340 10.1128/JVI.75.10.4692-4698.2001 PMC114223

[34] Meyers G, Thiel HJ (1996) Molecular characterization of pestiviruses. Adv Virus Res 47: 53–118. 8895831 10.1016/s0065-3527(08)60734-4

[35] Johns HL, Doceul V, Everett H, Crooke H, Charleston B, et al (2010) The classical swine fever virus N-terminal protease N(pro) binds to cellular HAX-1. J Gen Virol 91: 2677–2686. 20631090 10.1099/vir.0.022897-0

[36] Johns HL, Bensaude E, La Rocca SA, Seago J, Charleston B, et al (2010) Classical swine fever virus infection protects aortic endothelial cells from pIpC-mediated apoptosis. J Gen Virol 91: 1038–1046. 20007358 10.1099/vir.0.016576-0

[37] Tang Q, Guo K, Kang K, Zhang Y, He L, et al (2011) Classical swine fever virus NS2 protein promotes interleukin-8 expression and inhibits MG132-induced apoptosis. Virus Genes 42: 355–362. 21318239 10.1007/s11262-011-0582-z

