# Correction: The Untranslated Regions of Classic Swine Fever Virus RNA Trigger Apoptosis

**DOI:** 10.1371/journal.pone.0310393

**Published:** 2024-09-10

**Authors:** Wei-Li Hsu, Chung-Lun Chen, Shi-Wei Huang, Chia-Chen Wu, I-Hsuan Chen, Muthukumar Nadar, Yin-Peng Su, Ching-Hsiu Tsai

This Correction addresses an error in the preparation of Fig 1 of [1]. The originally published PK-15 panel in Fig 1 is incorrect and is a duplicate of lanes 1–4 of the 5’—Luc—3’ panel in Fig 4B. Here, the authors provide a revised Fig 1 using the correct image of the PK-15 gel from the original study.

**Fig 1:**
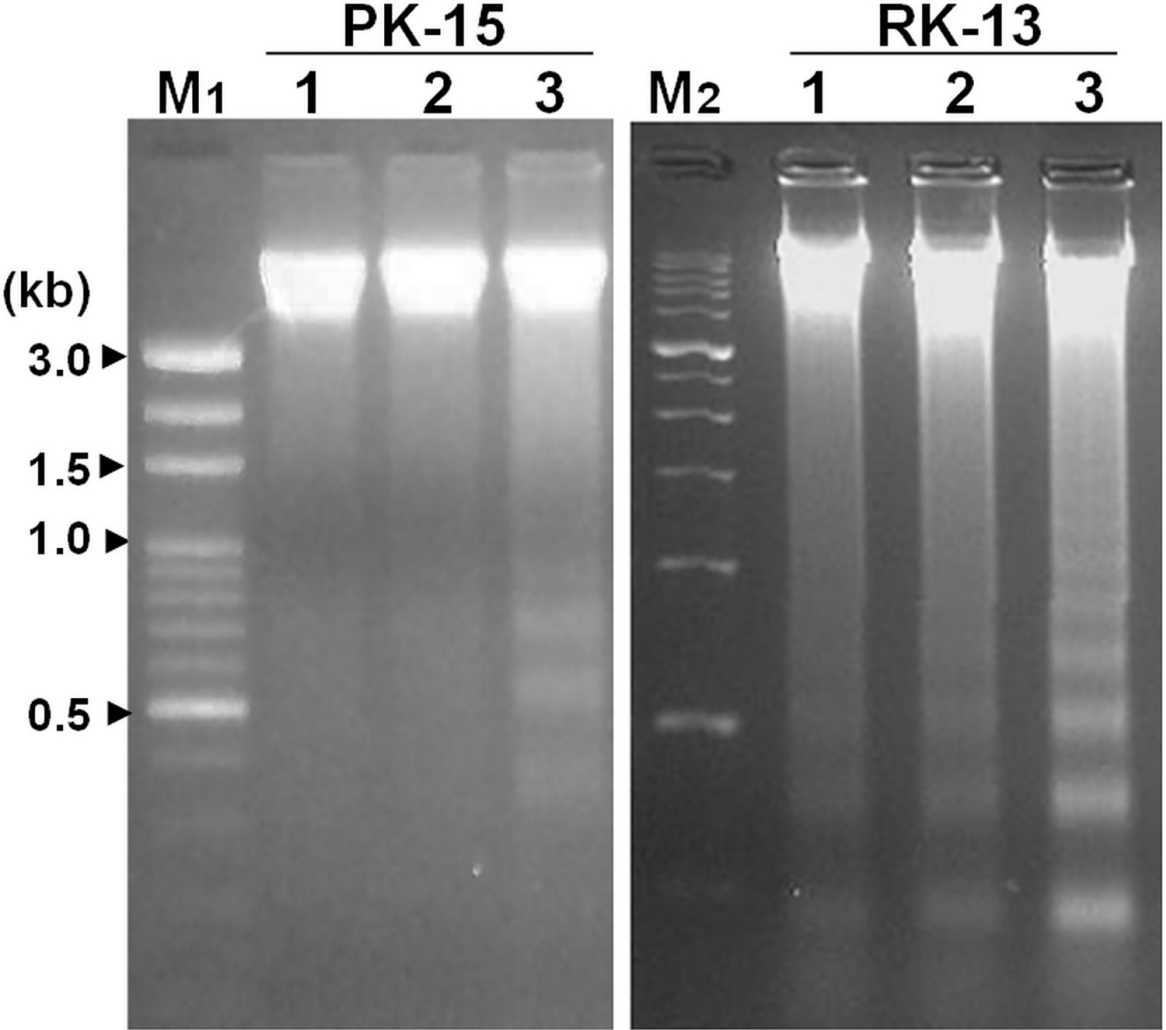
Transfection of CSFV UTR RNA triggers DNA laddering. Porcine kidney (PK-15) or rabbit kidney (RK-13) cells were mock transfected (lane 1), or transfected with *in vitro* transcribed luciferase RNA (lane 2), or chimeric luciferase-CSFV UTR RNA (5′UTR-Luc-3′UTR, lane 3) for 16 h. Total cellular DNA was harvested for the DNA laddering assay. M1, M2, and M were standard DNA markers with various size ranges.

The available images and individual-level quantitative data underlying the article’s figures are provided as Supporting Information (S1 and S2 Files).

The authors apologize for the error in the published article.

## Supporting information

S1 File Uncropped, original images underlying Figs 1, 2G, 3A, 3C, 4A–4D, 5A and 5B, 5D and 6. (PDF)

S2 File Individual-level quantitative data underlying Figs 2H, 3B and 5C. (ZIP)
