# Supplementary material for: Correction: The Untranslated Regions of Classic Swine Fever Virus RNA Trigger Apoptosis
Source: PLoS One. 2024 Sep 10;19(9):e0310393. doi: 10.1371/journal.pone.0310393 (PMC11386450; doi:10.1371/journal.pone.0310393)

Updated Fig 1

A wrong mage was used by mistake.

PK-15 should be replaced with this one

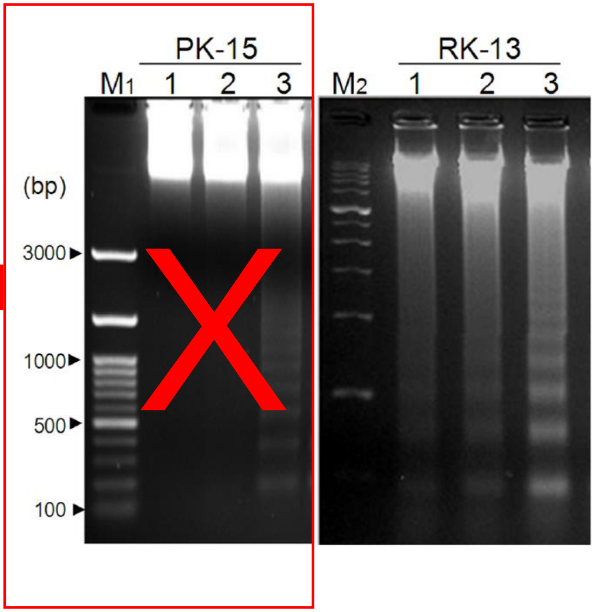

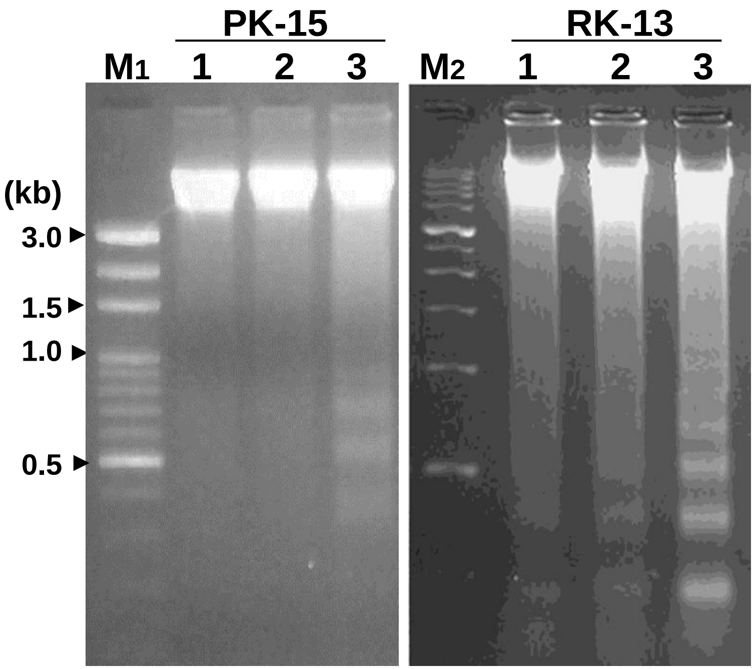

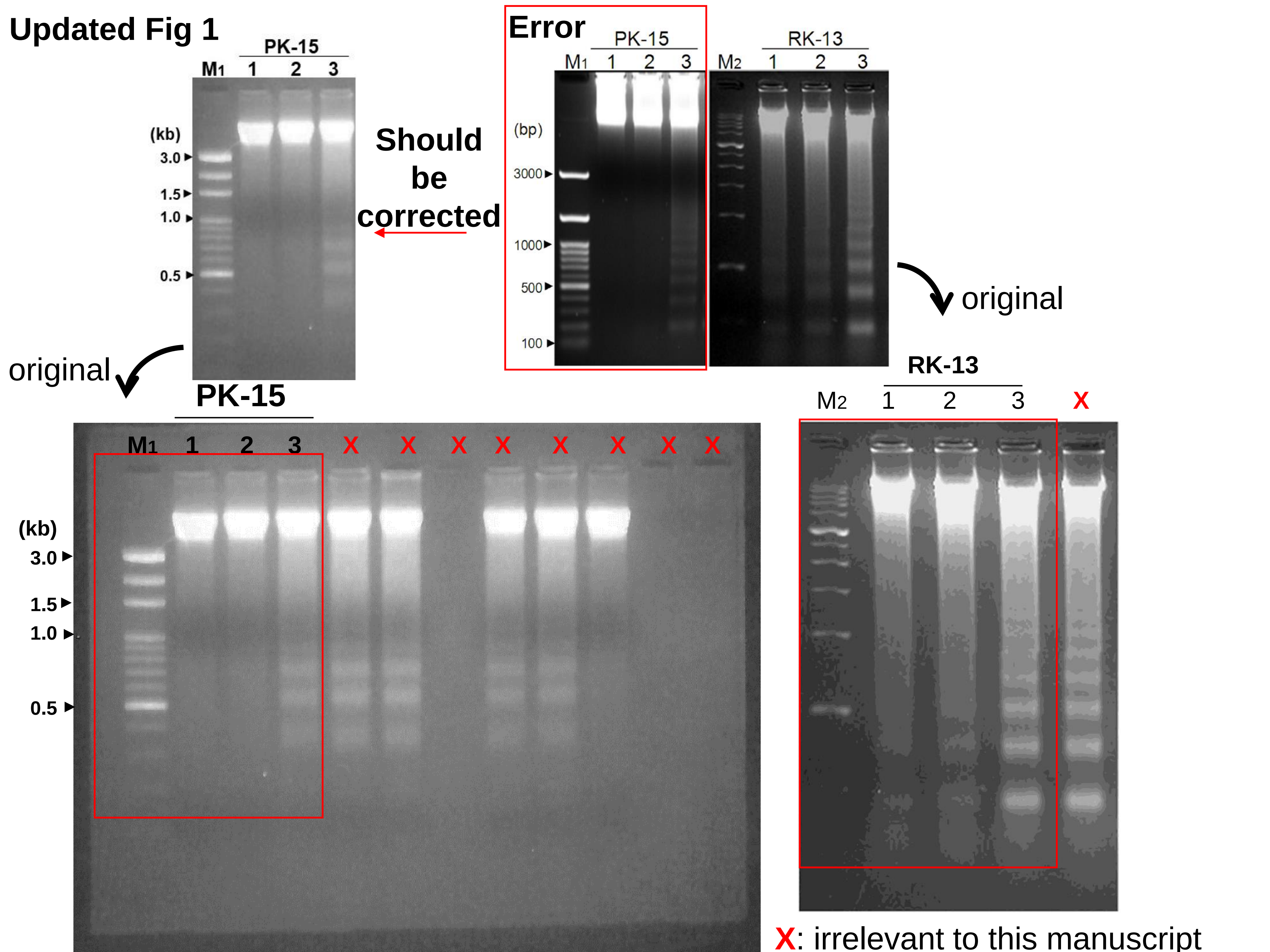
Updated Fig 1
PK-15
M1 1 2 3
(kb)
3.0
1.5
1.0
0.5
Should be corrected
Error
PK-15
M1 1 2 3
(bp)
3000
1000
500
100
RK-13
M2 1 2 3
original
original
PK-15
M1 1 2 3 X X X X X X X X
(kb)
3.0
1.5
1.0
0.5
RK-13
M2 1 2 3 X
X: irrelevant to this manuscript

Fig 2A-F

These images are original and uncropped

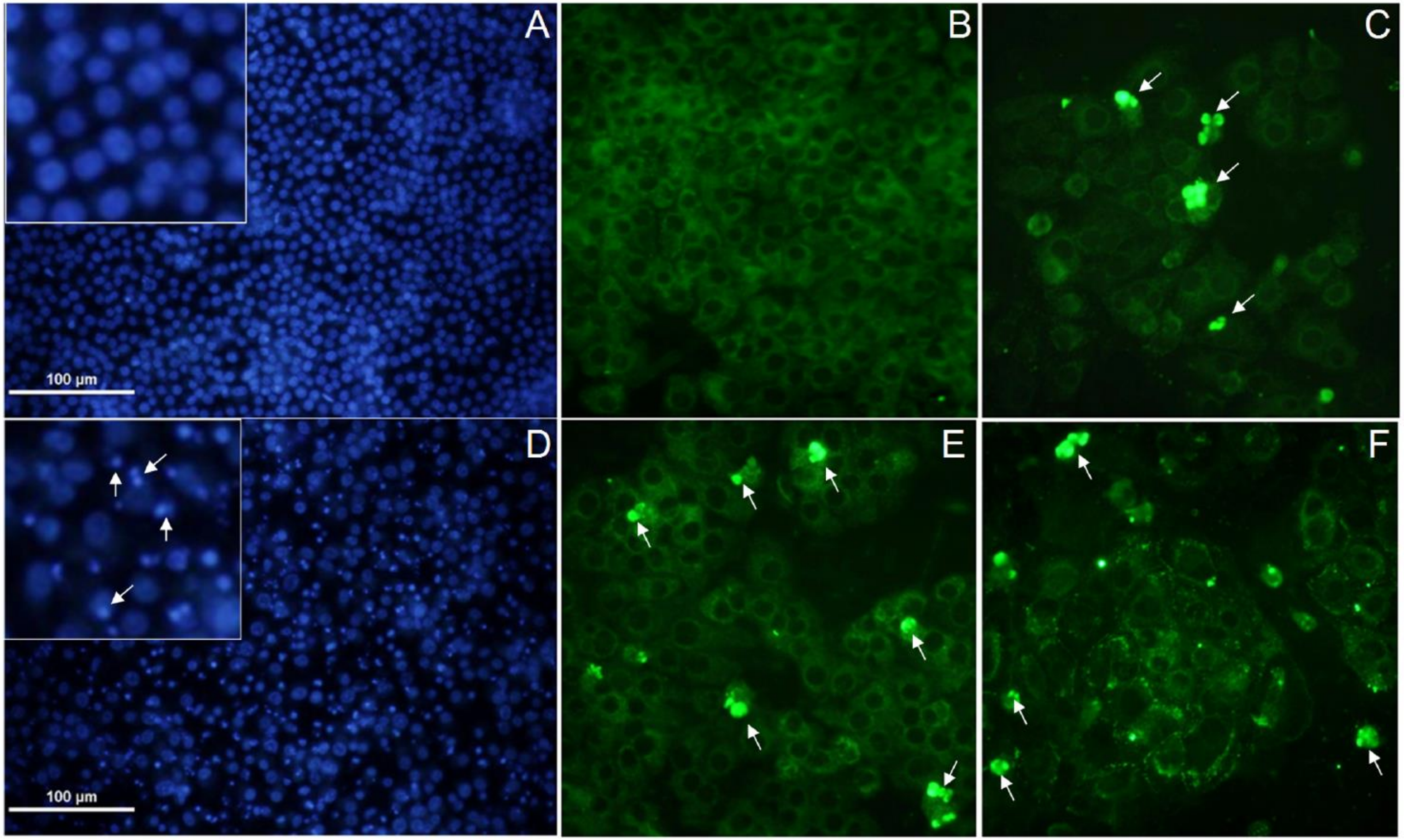

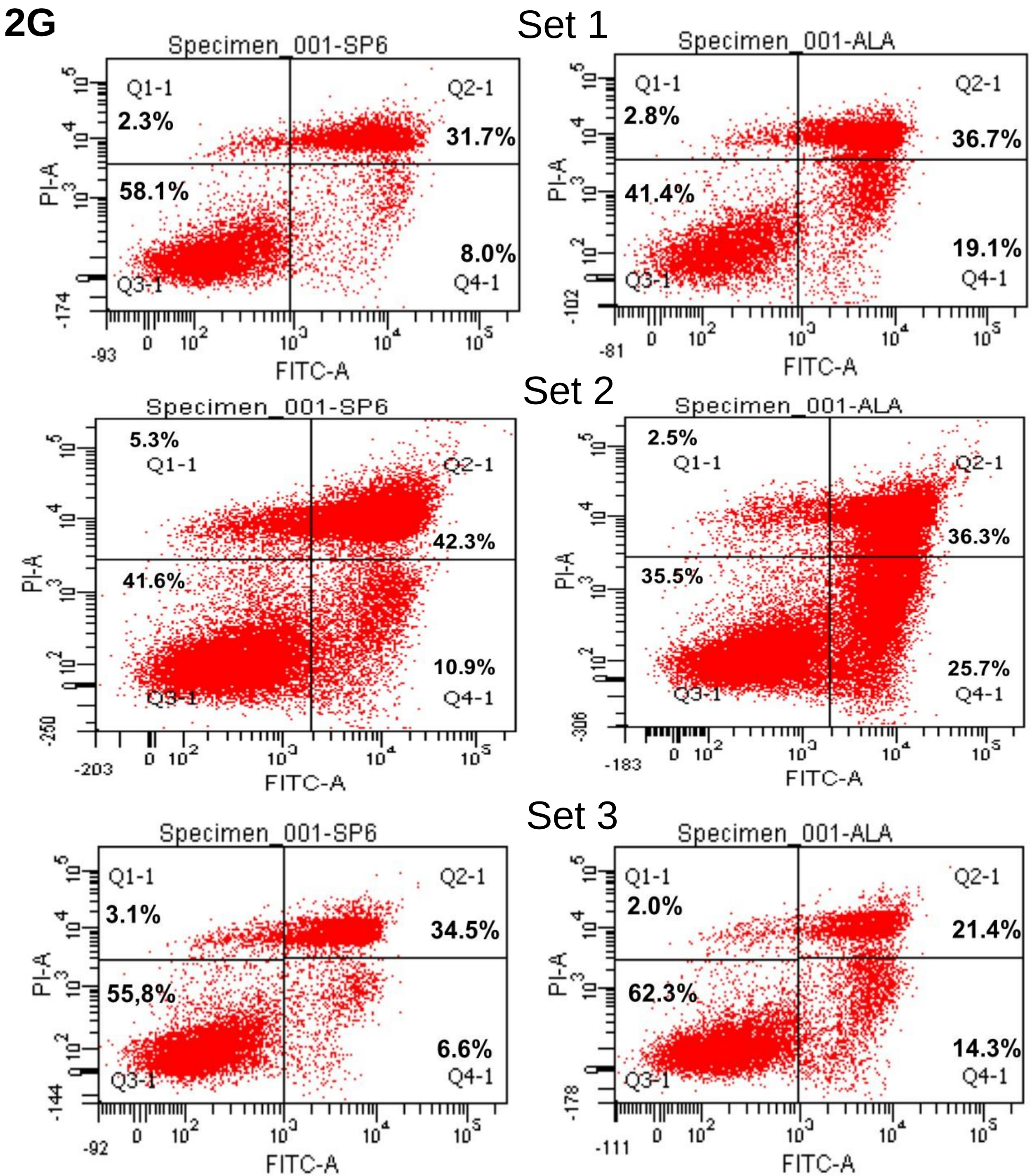

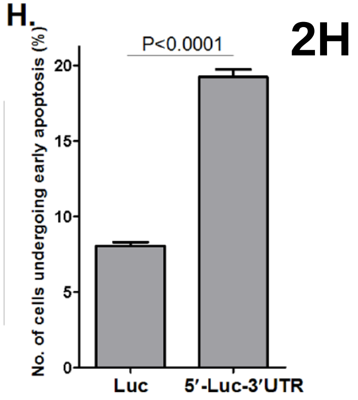

Original
(3 sets were retrieved)

R1+R4+R5	SP6	ALA
12/29(Q4-1)	8	19.1
12/13(Q4-1)	6.6	14.3
04/20(Q4-1)	10.9	25.7
AVG	8.50	19.7
SD	1.79	4.67
	LUC	5'-LUC-3'
AVG	8.5	19.7
SD	1.8	4.7

Fig 3A

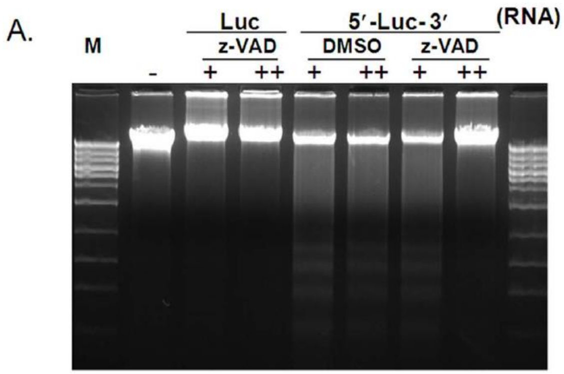

original

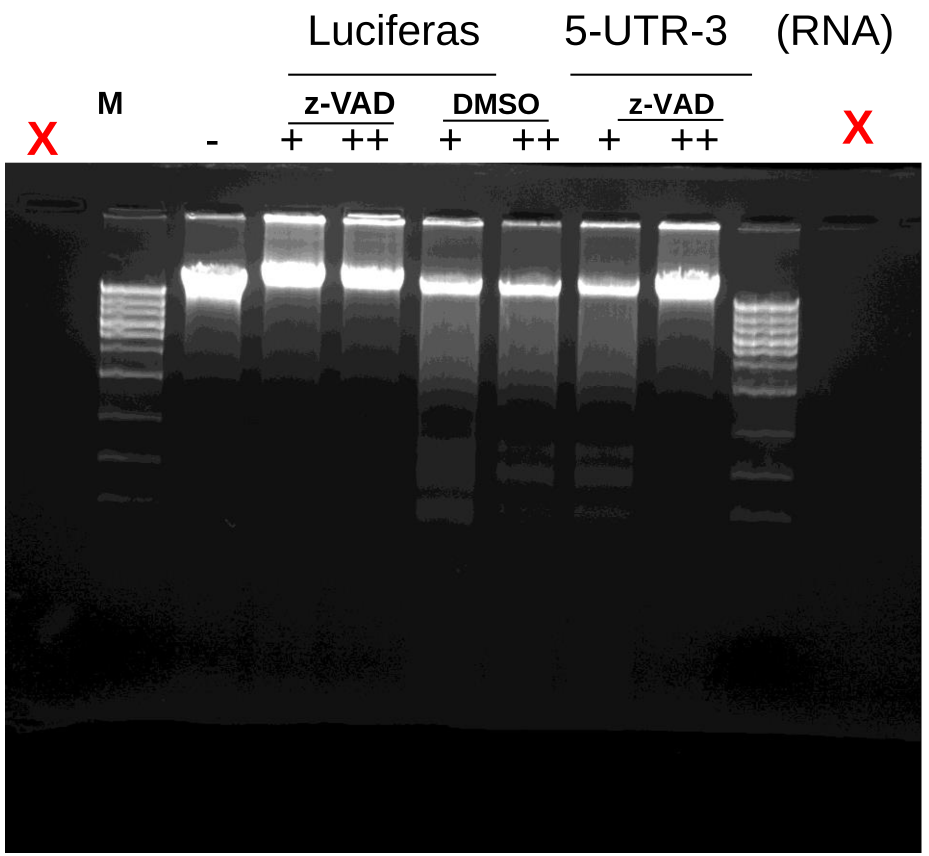

X: irrelevant to this manuscript

Fig 3B

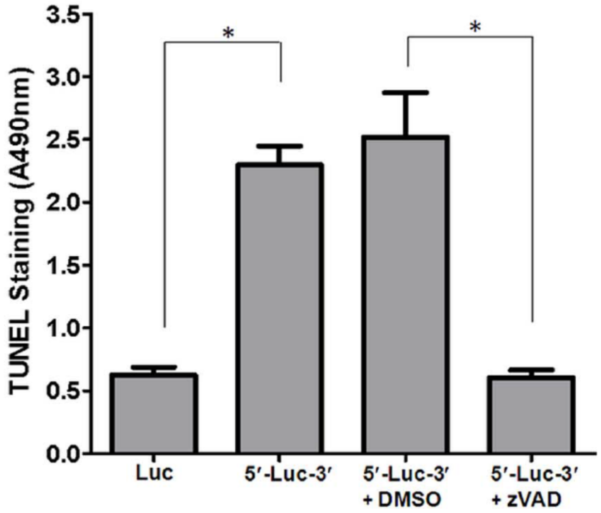

Original (only two sets were retrieved)

(Original Excel data enclosed)

32		mock	luc	5'-Luc-3'	5-luc-3+ DMSO	5-luc-3+ Z-VAD
33		0.972	0.439	2.173	2.036	0.417
34		1.492	0.348	1.959	2.539	0.333
35	avg.	1.232	0.394	2.066	2.288	0.375
36	sd	0.26	0.05	0.11	0.25	0.04
37						
38		mock	luc	5'-Luc-3'	5-luc-3+ DMSO	5-luc-3+ Z-VAD
39	average	1.23	0.39	2.07	2.29	0.38
40	sd	0.26	0.05	0.11	0.25	0.04
41						
42						
43						
44						
45						
46						
47						

Fig 3C

The two target protein signals were originated from one blot with two Abs.

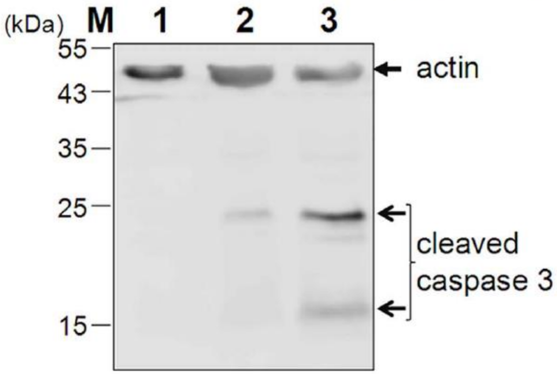

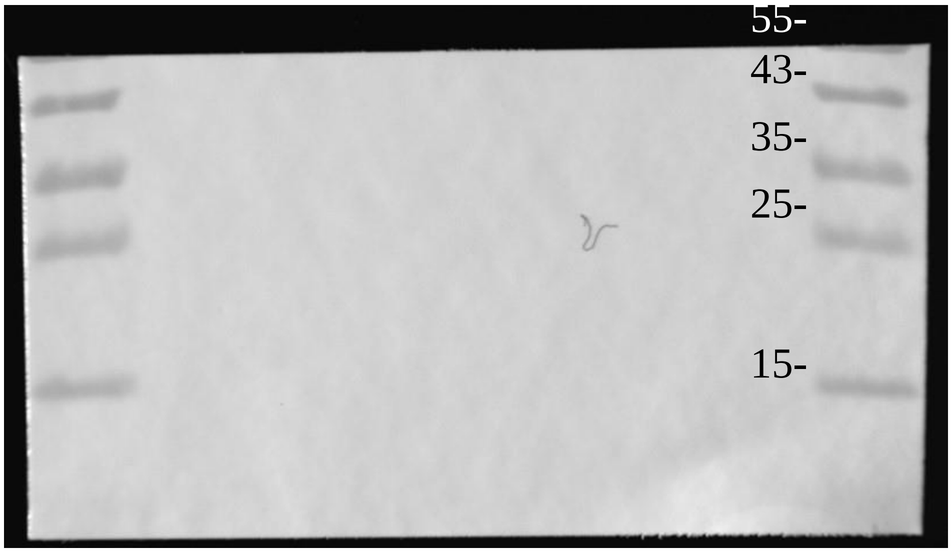

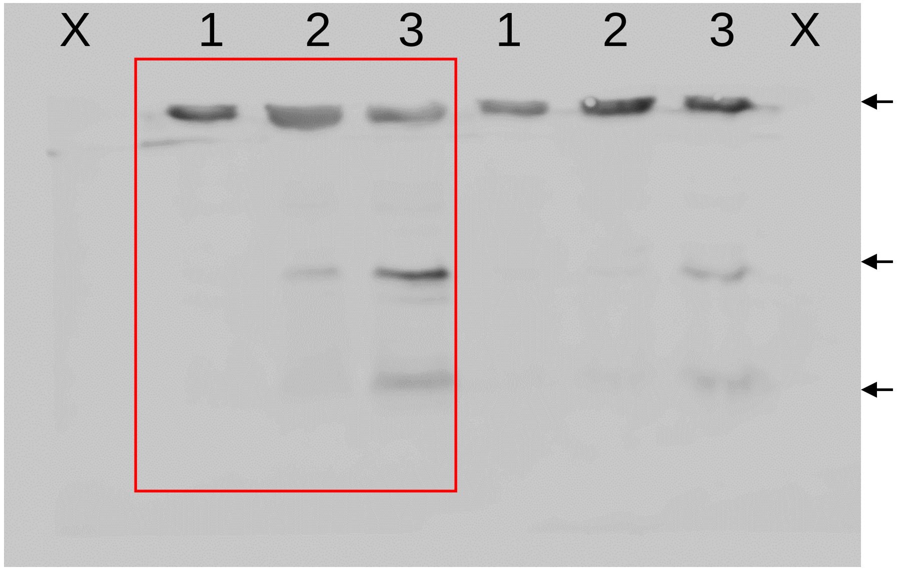

Bright field for markers

X: protein markers

Fig 4A

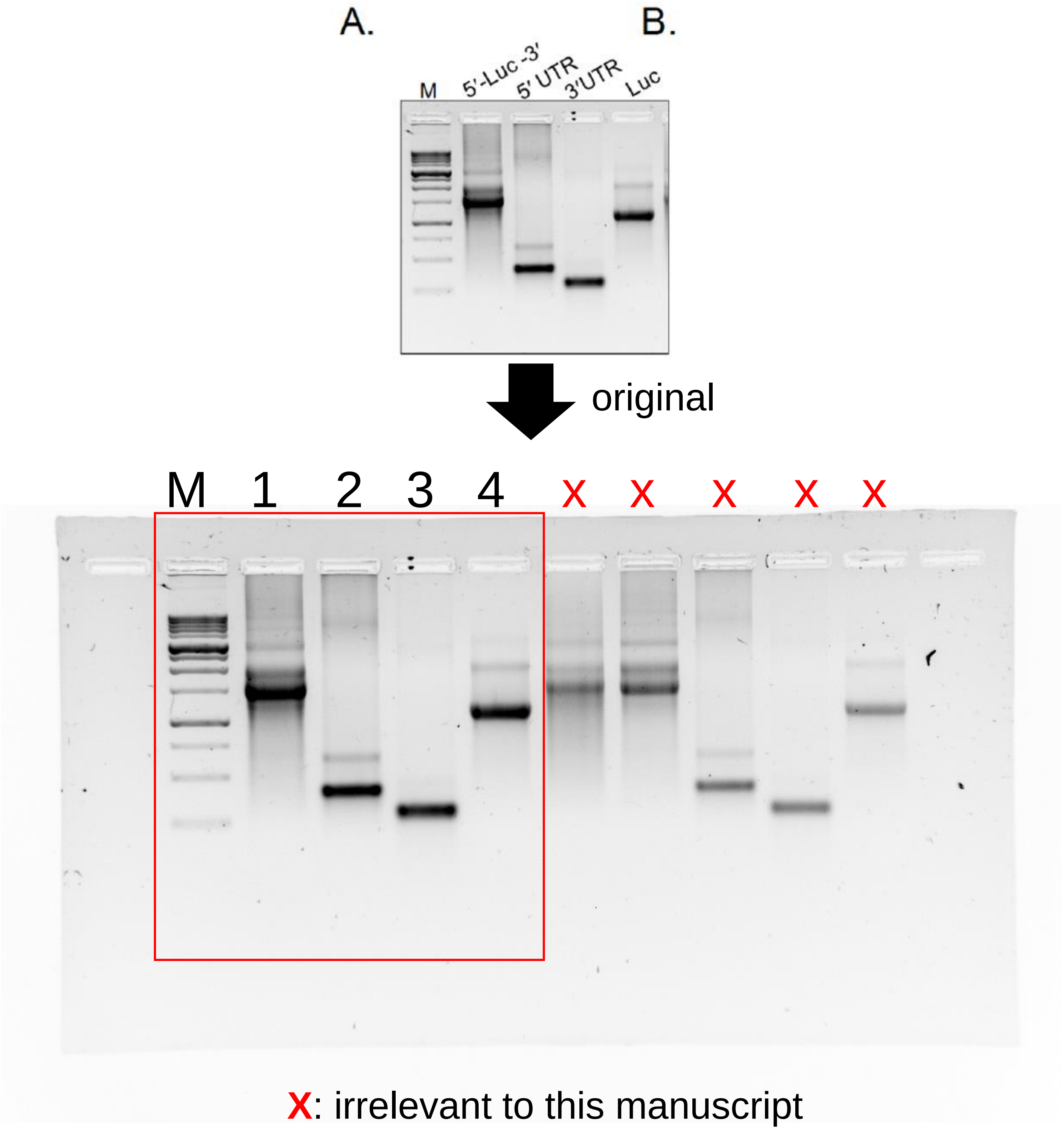

X: irrelevant to this manuscript

Fig 4B

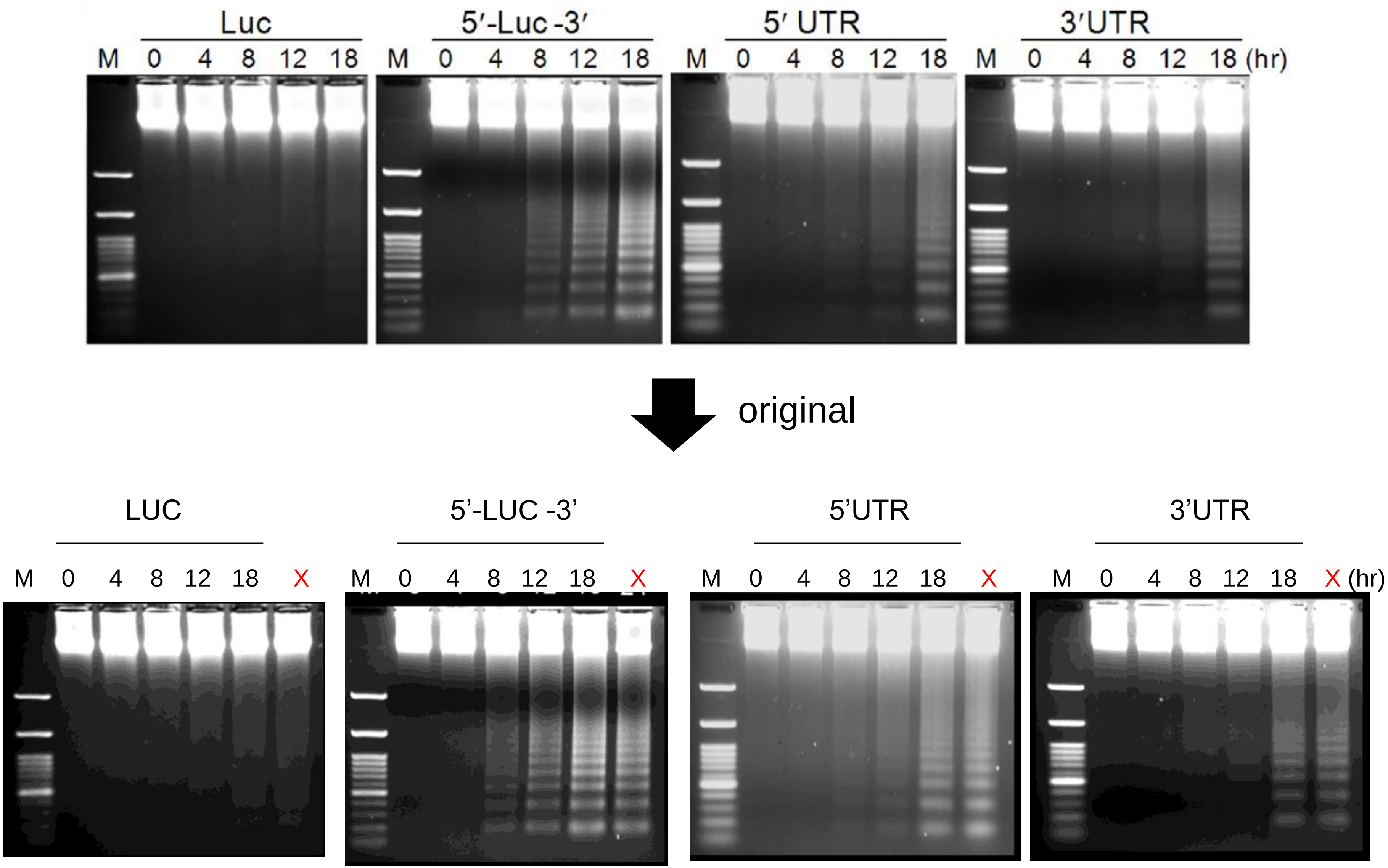

X: irrelevant to this manuscript

Fig 4C

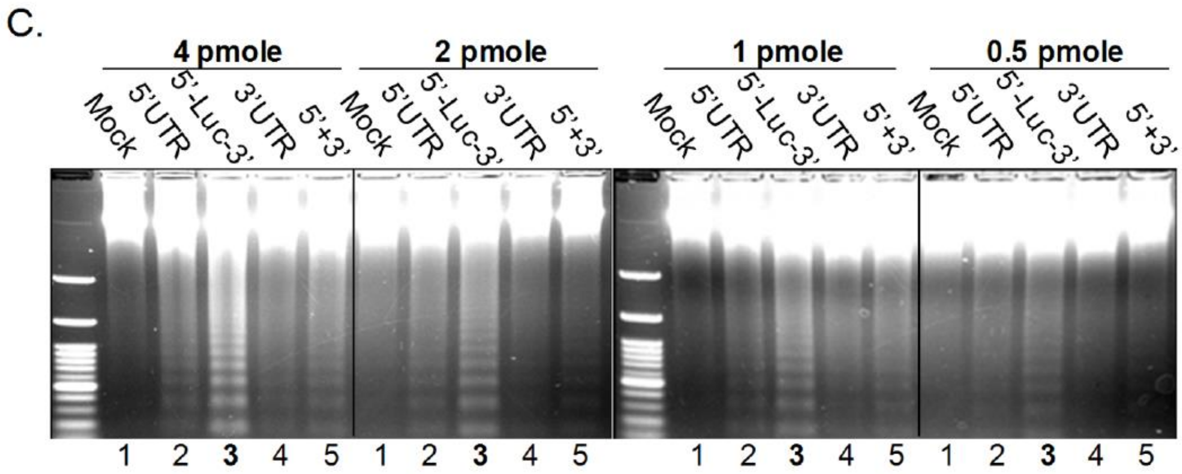
C.
4 pmole
2 pmole
1 pmole
0.5 pmole
Mock
5'UTR
5'-Luc-3'
3'UTR
5'+3'
1 2 3 4 5 1 2 3 4 5 1 2 3 4 5 1 2 3 4 5

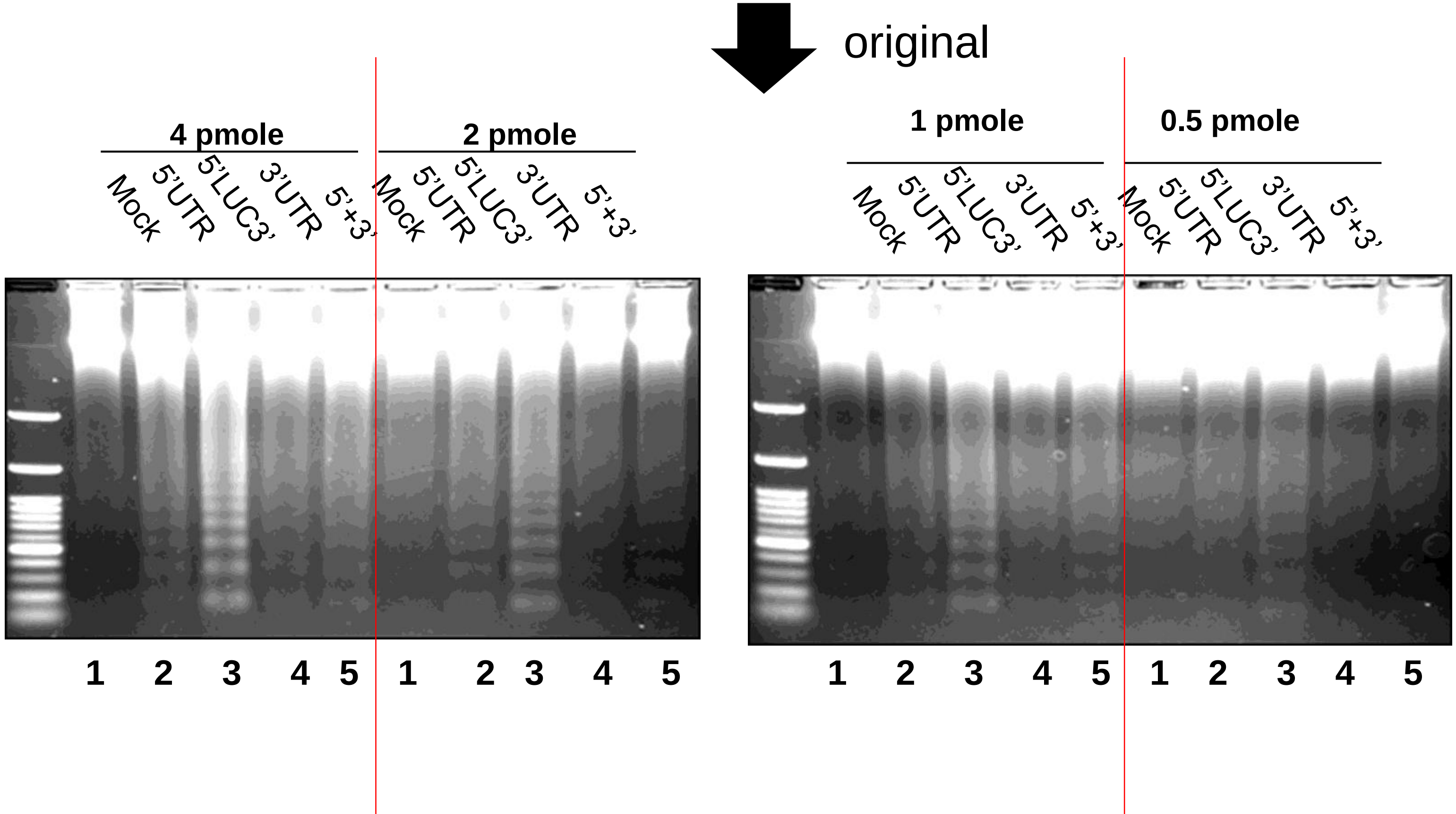
original
4 pmole
2 pmole
1 pmole
0.5 pmole
Mock
5'UTR
5'LUC3'
3'UTR
5'+3'
1 2 3 4 5 1 2 3 4 5 1 2 3 4 5 1 2 3 4 5

Fig 4D

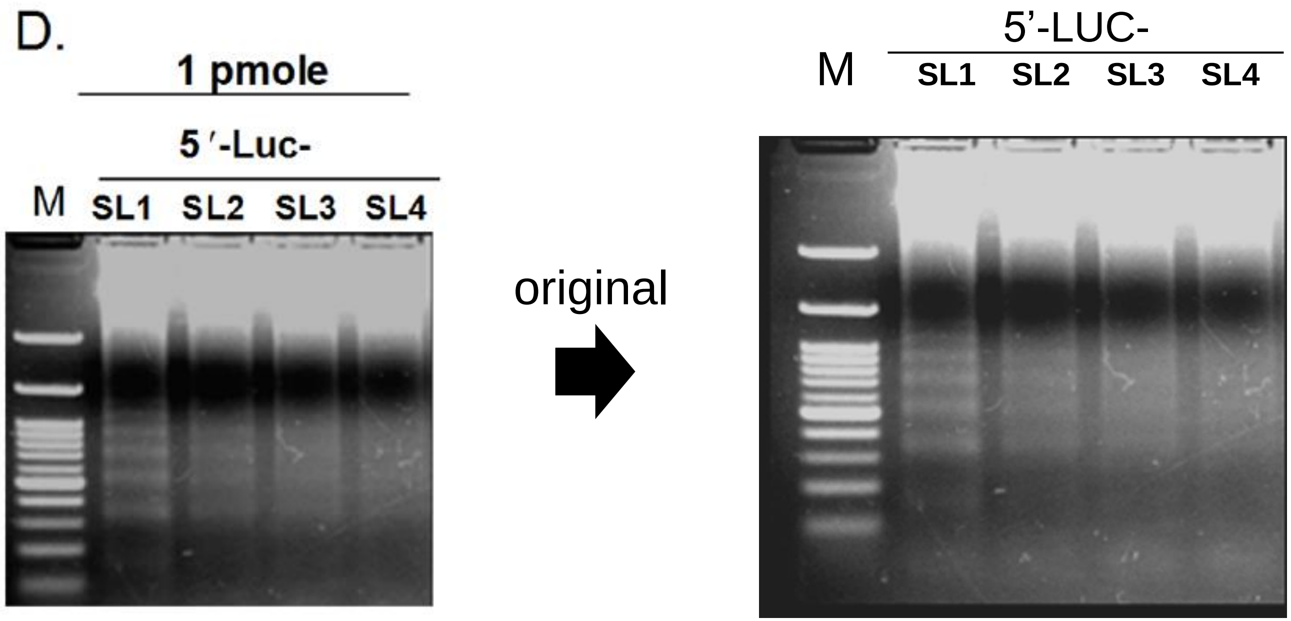

A.

Fig 5A

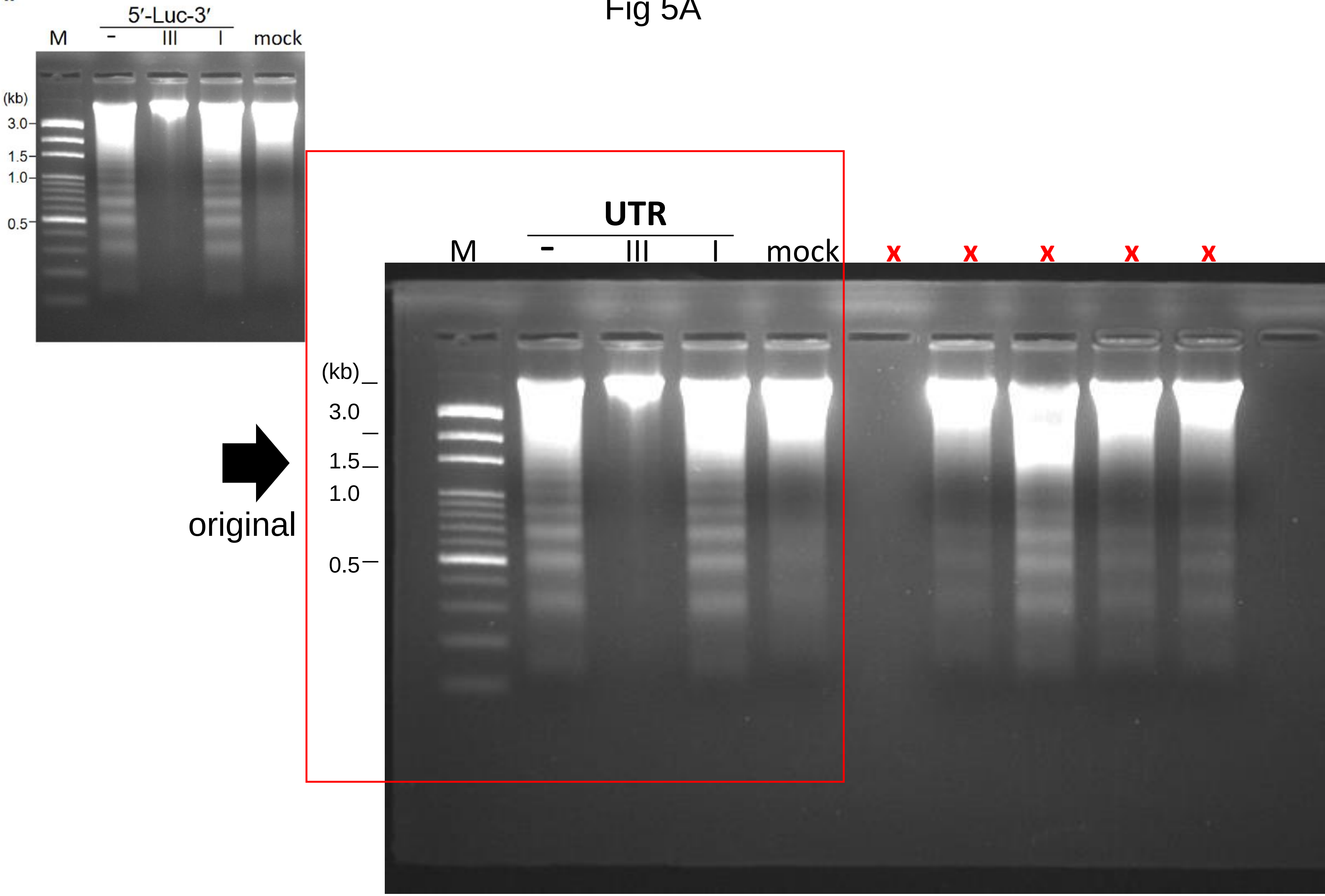

X: irrelevant to this manuscript

Fig 5B

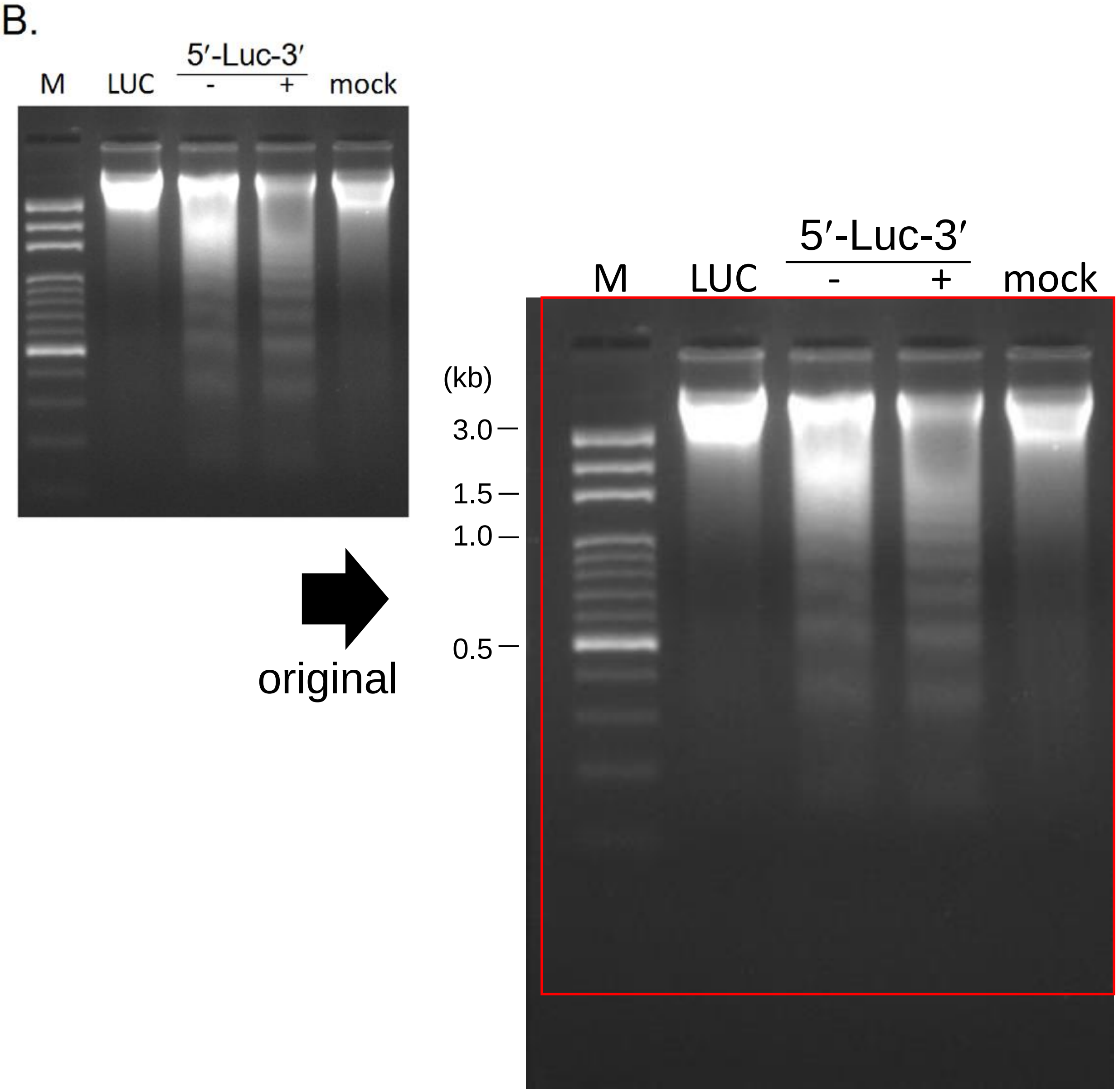

X: irrelevant to this manuscript

Fig 5C

C.

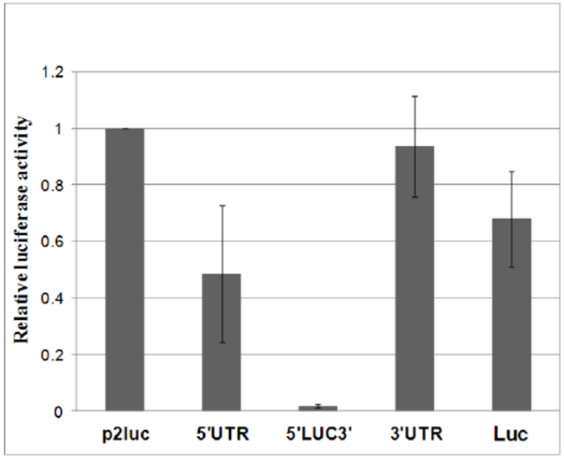

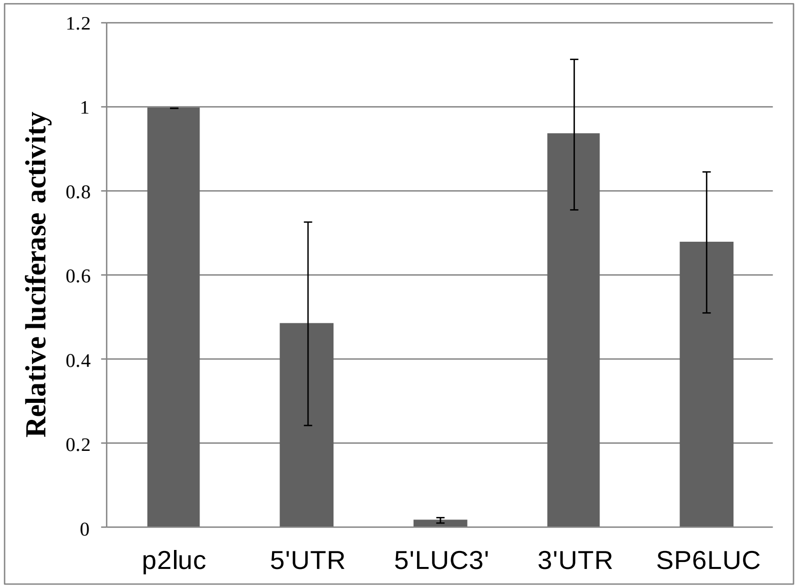

original

Original OD reads (ELISA)

	8月30日	9月7日	9月13日	stdev	avg
p2luc	1	1	1	0	1
5'UTR	0.267275	0.440277	0.745897	0.242354	0.484483
5'LUC3'	0.017301	0.011173	0.022184	0.005517	0.016886
3'UTR	0.753906	0.945129	1.110696	0.178549	0.936577
SP6LUC	0.521023	0.658711	0.857159	0.168981	0.678964

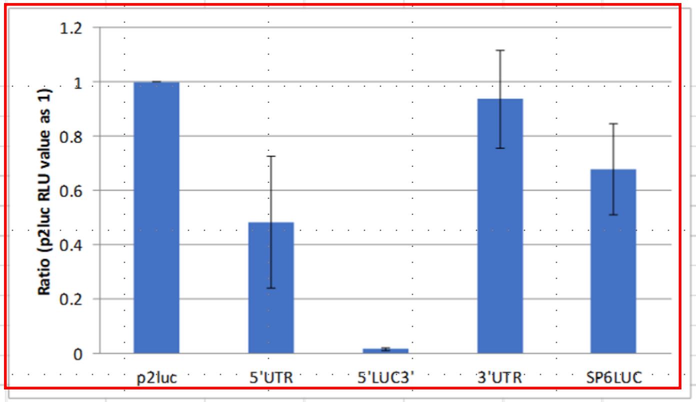

Fig 5D

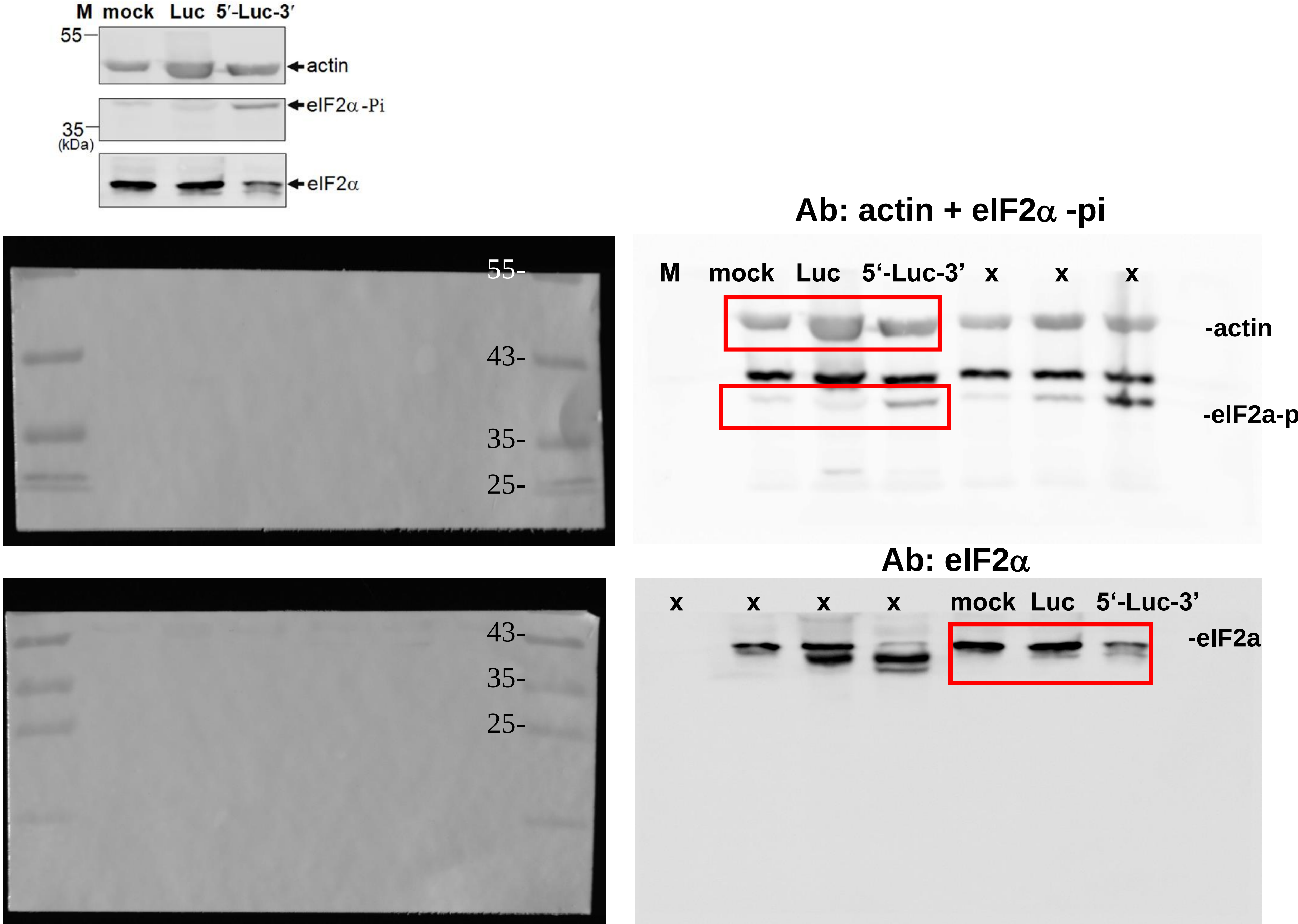
M mock Luc 5′-Luc-3′
55
actin
eIF2α -Pi
35
(kDa)
eIF2α
Ab: actin + eIF2α -pi
55-
43-
35-
25-
M mock Luc 5‘-Luc-3’ x x x
-actin
-eIF2a-p
Ab: eIF2α
43-
35-
25-
x x x x mock Luc 5‘-Luc-3’
-eIF2a

Fig 6

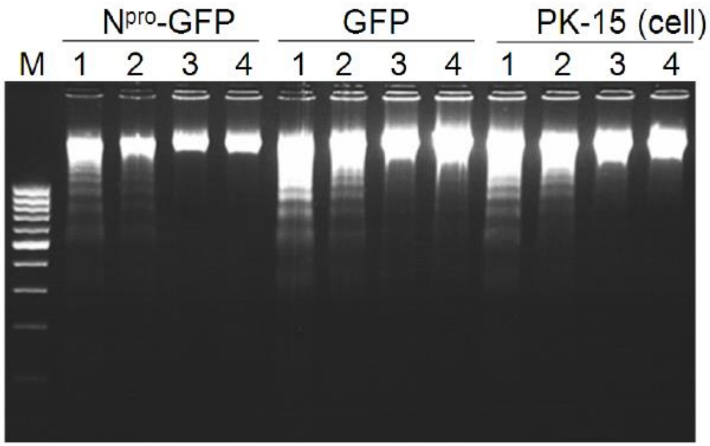

original

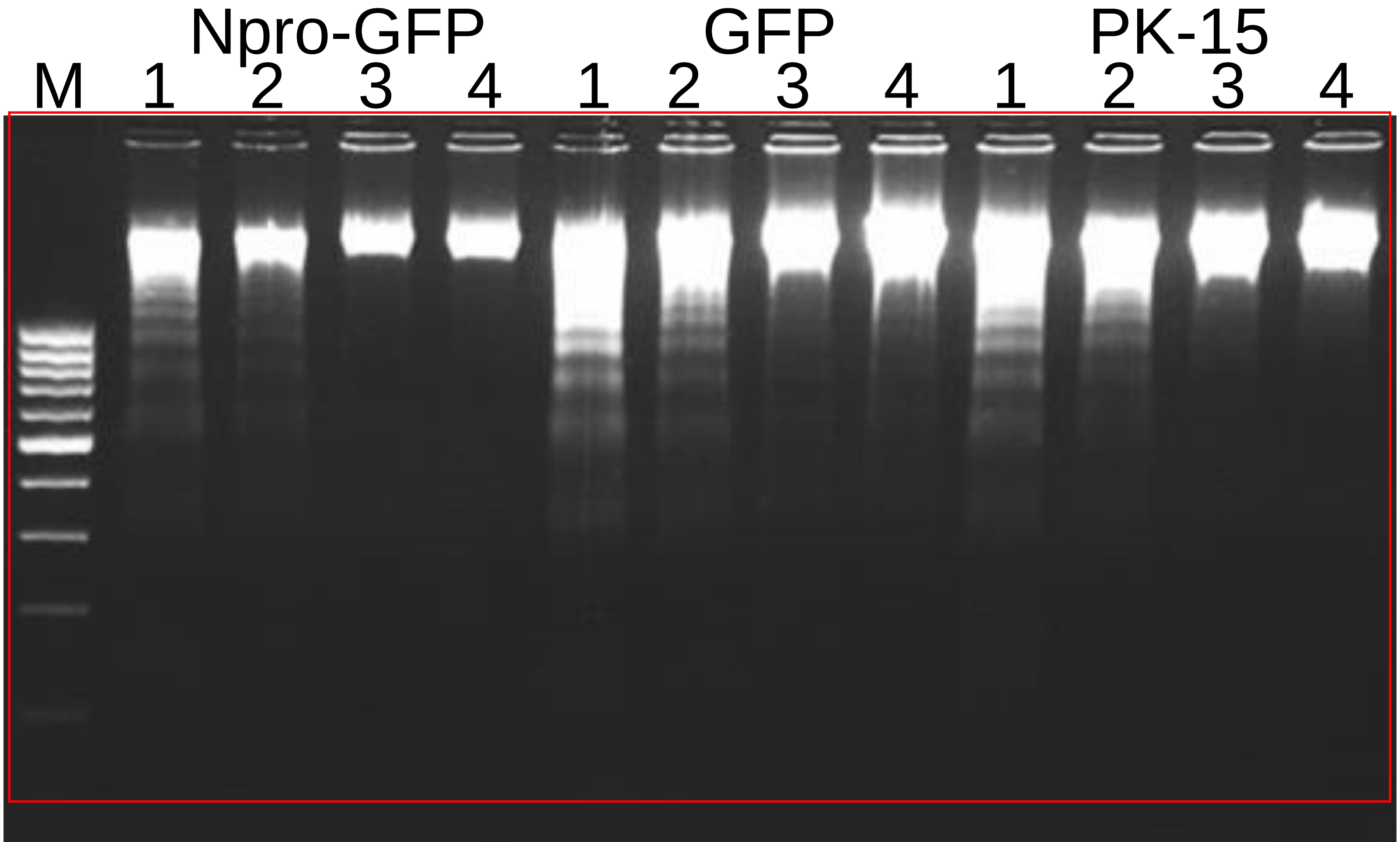

Supplement: S1 File — (PDF) [file pone.0310393.s001.pdf]
